# What Users Say About Reimbursable Digital Therapeutics in Germany: Large-Scale App Store Review Analysis Using a Large Language Model

**DOI:** 10.2196/92415

**Published:** 2026-10-08

**Authors:** Benjamin Kinast, Henrik Rohde, Björn Schreiweis, Hannes Ulrich

**Affiliations:** 1 Institute for Medical Informatics and Artificial Intelligence, Kiel University and University Hospital Schleswig-Holstein, Kaistrasse 101, Kiel, DE-24105, Germany, +49 431 500 31611; 2 Department of Medical Informatics, Institute for Community Medicine, University Medicine Greifswald, Greifswald, Germany

**Keywords:** mobile health, mHealth, eHealth, apps, digital therapeutics, large language model, LLM, applications

## Abstract

**Background:**

In 2019, Germany introduced a unique regulatory framework for digital therapeutics (DTx), termed digital health applications (DiGAs) in Germany, with the goal of integrating evidence-based DTx into statutory health care. DTx are eligible for reimbursement by statutory health insurance if manufacturers demonstrate positive health care effects, such as improved health status or health literacy, in a controlled study setting. Although regulatory evaluation primarily relies on manufacturer-conducted studies, these studies do not fully capture how users experience and use DiGAs in everyday life.

**Objective:**

The study aims to systematically characterize user experiences with reimbursable DiGAs by analyzing the sentiment and thematic content in publicly available app store reviews at scale to identify recurring patterns of positive and negative feedback across regulatory-relevant quality dimensions.

**Methods:**

All DiGAs with publicly available mobile app reviews were identified via the official German Federal Institute for Drugs and Medical Devices (BfArM) directory. User reviews were extracted from the German Apple App Store and Google Play Store using a tailored Python script. Reviews were then processed using GPT-4o, which extracted up to 5 core statements per review and assigned each a sentiment label (positive, neutral, or negative) and one of ten predefined thematic categories to each statement: content, technology, cost and reimbursement, login and registration, prescription and approval, user experience and design, support, tracking and documentation, effectiveness, and overall impression, all derived from the regulatory and quality criteria applicable to DiGA certification. Classification quality was assessed through manual validation, in which each of the 3 authors independently reviewed one-third of all model outputs.

**Results:**

In total, 44 mobile DiGAs were included and analyzed. After data extraction and cleansing, the final dataset comprised 4328 reviews containing at least one interpretable statement, resulting in 9439 interpretable statements. A systematic validation of the automated classification demonstrated exceptionally high model performance, with 99% accuracy for sentiment classification (*F*_1_-scores of 1.00 for positive and 0.99 for negative categories) and 95% accuracy for category classification (average *F*_1_-score of 0.95). While the categories overall impression (1233/1431, 86.2% positive) and effectiveness (1440/1609, 89.5% positive) received particularly positive feedback, users commented most negatively on the login and registration process (263/280, 93.9% negative) and technology-related aspects (563/668, 84.3% negative).

**Conclusions:**

User feedback on reimbursable DiGAs is predominantly positive, particularly regarding perceived effectiveness, overall impression, and content. However, recurring criticism of login and registration processes, technical reliability, and prescription and approval procedures reveals persistent barriers to access and use. As many of these aspects are prerequisites rather than peripheral convenience issues, they should be treated as essential implementation requirements. Analyzed at scale with large language model-based methods, publicly available app store reviews can complement formal evaluation by providing user-centered real-world evidence for postmarket monitoring and regulatory quality improvement.

## Introduction

In 2019, Germany introduced a unique regulatory framework for digital therapeutics (DTx) known as digital health applications (DiGAs), with the goal of integrating evidence-based DTx into routine care [1,2]. Under the Digital Healthcare Act (Digitale-Versorgung-Gesetz) [3], DiGAs can be prescribed by physicians and reimbursed by statutory health insurance if they are listed in an official directory maintained by the German Federal Institute for Drugs and Medical Devices (BfArM) [4]. The directory covers DTx across a range of indications, such as mental and behavioral disorders, musculoskeletal disorders, metabolic disorders, and disorders of the nervous system. This initiative aims to improve access to innovative health care technologies while ensuring their safety, quality, and effectiveness.

To qualify for inclusion, DTx must meet clinical, legal, and technical requirements, including demonstrable benefits to patient care or care structures as defined in section 139e of the German Social Code, Book V (SGB V) [3]. These so-called positive health care effects refer to either a medical benefit or to patient-relevant improvements in structures and processes. A medical benefit is defined as an effect such as an improvement in health status, a reduction of disease duration, prolonged survival, or increased quality of life. Patient-relevant structural or procedural improvements may include better coordination of the care process, easier access to health care services, or the promotion of health literacy [5]. To facilitate timely integration into routine care, the law also additionally introduced a fast-track procedure that allows provisional listing for up to 12 months while manufacturers gather evidence of positive health care effects [6,7].

Although regulatory evaluation rests primarily on manufacturer-conducted clinical studies [8,9], such studies are not designed to capture the patient-centered dimensions that determine whether a DiGA is adopted and used in everyday life [10]. For the German DiGA context specifically, it has been argued that acceptance, perceived usefulness, and usability are decisive for sustained use, yet genuine user-centeredness is not yet firmly embedded in DiGA development [11]. Usability, acceptability, and the practical feasibility of integrating an app into daily routines are key determinants of sustained engagement, yet they are only partially addressed during development and approval. To date, the actual use and adherence to a DTx have been identified as a critical but undersupported determinant of effectiveness, for which clinical practice still lacks established assessment tools [12]. Low uptake and prescription rates further underline that factors beyond clinical efficacy shape whether DiGAs reach and benefit patients [13]. As several studies emphasize, user reviews of mobile health (mHealth) apps can reveal valuable insights into real-world usability and recurring quality concerns. All these dimensions are directly relevant to the DiGA approval framework [14-17]. Analyzing user-generated feedback can therefore offer a scalable, real-world complement to clinical evidence and may surface practical barriers to adoption and sustained use.

Importantly, the regulatory framework itself recognizes the relevance of patient perspectives. Section 139e SGB V requires that the outcomes of digital health apps include patient-reported health status during usage as part of mandatory success monitoring [3]. This emphasis is mirrored in the DiGA guideline (DiGA-Leitfaden), which explicitly permits manufacturers to demonstrate positive health care effects using comparative quantitative methods drawn from the social or behavioral sciences, provided validated instruments are used [18]. This institutional acknowledgment underscores that patient experience has become a regulatory concern in the DiGA framework. App store reviews provide spontaneous, large-scale, real-world feedback across the entire DiGA landscape in a way that standardized instruments do not. While they cannot replace validated patient-reported outcome instruments, their systematic analysis can potentially reveal how DiGAs are experienced in everyday use.

To date, however, patient feedback on German DiGAs has received limited systematic attention. Uncovska et al [19] analyzed app store reviews of mHealth apps in Germany, comparing regulated DiGAs with nonregulated consumer apps using BERTopic modeling and sentiment analysis. Their work showed that DiGAs received more favorable contemporary ratings than nonregulated apps. Positive themes included customer service, personalization, and ease of use, while software bugs and cumbersome registration processes were frequent complaints across both app types. Their analysis, however, covered only 15 DiGAs and relied on data-driven topic categories that do not map onto the regulatory dimensions against which DiGAs are formally assessed. Furthermore, it was framed primarily as a comparison between regulated and nonregulated apps rather than a focused account of the user experience with DiGAs themselves.

Internationally, app store reviews have been used to examine user experience with health apps in various contexts, ranging from consumer health apps to regulated prescription DTx in the United States [20,21]. This leaves a clear gap, as to our knowledge, there is no systematic, large-scale account of how users evaluate the full range of listed DiGAs along the dimensions that are central to their regulatory assessment. To address this gap, this study analyzes publicly available DiGA user reviews from the German Apple App Store (iOS) and the Google Play Store (Android) for all approved and provisionally approved mobile DiGAs as of February 25, 2025.

Using a regulation-informed category scheme and large language model (LLM)–assisted classification, we aim to characterize how users evaluate DiGAs across dimensions relevant to regulatory assessment and to identify where regulatory expectations and lived user experience may converge or diverge.

## Methods

### Data Identification and Extraction

Data collection involved 2 stages: first, we identified all mobile DTx listed by the BfArM as of February 25th, 2025. Second, we used Selenium-based Python scripts (Version 4.29.0) to extract user reviews from the German Apple App Store (iOS) and the Google Play Store (Android). Extracted data included review content, ratings, dates, developer responses, and app metadata, all stored in structured JSON format.

### Category Development and Regulatory Grounding

The thematic category scheme was developed using a regulatory-driven approach. Categories were derived from legal and regulatory requirements governing DiGAs in Germany, with the aim to ensure regulatory relevance and interpretability of user-reported feedback. Specifically, we systematically reviewed the SGB V, the Digital Health Applications Regulation (DiGAV), and the official BfArM DiGA guideline [5,18]. From these sources, we identified recurring normative evaluation dimensions relevant to DiGA approval, quality assurance, and postmarket monitoring. These include requirements related to content quality, technical stability, usability, effectiveness, reimbursement, and user experience. Based on this review, 10 high-level categories were defined, and each was mapped to one or more regulatory criteria to ensure traceability between app-user feedback and legally relevant quality dimensions (Table 1). The framework was developed deductively, as the study aimed to assess user feedback against the quality dimensions that are legally binding for reimbursable DiGAs. During the human validation, the authors additionally assessed whether statements addressed aspects not covered by the framework.

**Table 1.** The 10 feedback categories, the user-reported content they capture, and their regulatory grounding.

Category	Content captured	Regulatory basis
Content	Informational, educational, and therapeutic material; evidence-based scientific soundness, up-to-dateness, readability	section 139e (2) SGB V^a^; section 5 (8) DiGAV^b^
Technology	Technical stability, software reliability, device compatibility, bugs, and system failures	section 139e (2) SGB V; section 5 (2) DiGAV; BfArM^c^ guideline
Cost and reimbursement	Perceived cost; reimbursement through statutory health insurance	section 134 (1) SGB V
Login and registration	Initial access; ease of login and registration	section 5 (5) DiGAV
Prescription and approval	Obtaining or redeeming access codes; prescription, insurer approval, and diagnosis requirements	BfArM guideline (Sections 3.2.2, 2.1.2.2)
User experience (UX) and design	User interface, usability, visual, and design aspects (overall interaction beyond initial access)	section 5 (5) DiGAV
Support	Availability, responsiveness, and quality of customer or therapeutic support	BfArM guideline (Section 3.6.2.5)
Tracking and documentation	Documentation of symptoms, nutrition, activities, or behaviors	section 139e (13) SGB V (effective Jan 1, 2026)
Effectiveness	Perceived medical benefit, self-management, health literacy, and therapy adherence	section 139e (13) SGB V; section 8 (2)-(2) DiGAV
Overall impression	Overall subjective rating, perceived utility, and user trust	section 139e (13) No. 2 SGB V

a SGB V: German Social Code, Book V.

b DiGAV: Digital Health Applications Regulation.

c BfArM: German Federal Institute for Drugs and Medical Devices.

A more detailed overview of the legal sources and their mapping to categories is provided in Multimedia Appendix 1.

### Model-Based Sentiment and Category Assignment

To systematically assess user sentiment, we developed a custom Python script to automate the classification process using the GPT-4o API provided by OpenAI (steps 1‐3). GPT-4o was selected since previous research has shown that GPT-based LLMs can achieve strong zero-shot performance in sentiment classification of user-generated texts and online reviews [22]. Each user review was processed individually using a carefully structured prompt designed to guide the model toward consistent and domain-specific classification (Multimedia Appendix 2). The model was instructed to adopt the role of an expert in evaluating user experiences with DiGAs. The prompt was developed iteratively before the full-scale analysis. Initial prompt versions were piloted on a heterogeneous subset of reviews and refined through repeated inspection of ambiguous, inconsistent, or insufficiently specific model outputs. Revisions focused on clarifying category definitions, decision rules for mixed sentiment, and the required structured output format. This iterative prompt-development approach follows emerging methodological guidance emphasizing prompt specification, testing, and refinement for reliable LLM-supported classification tasks [23]. Within this role, it performed the following multistep tasks:

- Extract up to 5 core statements from each user review.
- Assign each statement a sentiment label (positive, neutral, or negative).
- Assign each statement to exactly one of the 10 predefined thematic categories derived from the legal and quality criteria for DiGAs (Multimedia Appendix 1).
- Merge statements that refer to the same theme and sentiment into a single, summarized expression.
- Avoid repeating partial aspects of the same issue across multiple statements.
- Limit each category to one statement per review, unless clearly distinct subtopics are present.
- Exclude overall evaluations if the review indicates that the app could not be used (eg, due to failed registration or missing insurance approval).

The model was provided with a fixed list of 10 valid categories, accompanied by specific guidance on ambiguous cases. For example, access issues after prescription or insurer approval were to be categorized as login and registration, not prescription and approval. User reviews may contain several distinct evaluative aspects. Extracting up to 5 core statements allowed the analysis to capture this multidimensionality while maintaining a consistent upper limit across reviews and preventing unusually long reviews from disproportionately influencing the dataset. Finally, the model was instructed to return its output in a standardized, structured format with numbered statements, each containing 3 elements: the *extracted statement*, *sentiment label*, and *assigned category*. A representative example was included in the prompt to enforce consistent formatting and interpretation. In order to assess whether an unsupervised topic-modeling approach could provide a suitable alternative to the predefined, prompt-based classification [24], we additionally conducted an exploratory BERTopic analysis following Uncovska et al [19]. BERTopic was applied to the complete corpus of user reviews to identify recurring themes without imposing predefined categories. The resulting topic clusters were assessed with respect to thematic coherence, clinical interpretability, and correspondence with the 10 predefined DiGA evaluation categories. In addition, we considered whether the approach could adequately represent multiple distinct issues within a single review. This exploratory comparison informed the selection of the final analytical approach. In the following processing steps, the script parsed the structured model output into a tabular dataset containing the extracted statements and their corresponding sentiment labels and thematic categories (steps 4‐5). All results were exported in a standardized CSV format for further manual validation (step 6). By assigning the model a clearly defined expert perspective and domain logic, we ensured that the sentiment analysis aligned with both linguistic consistency and health service evaluation standards. A fictitious example of this classification process is shown in Figure 1.

**Figure 1.**
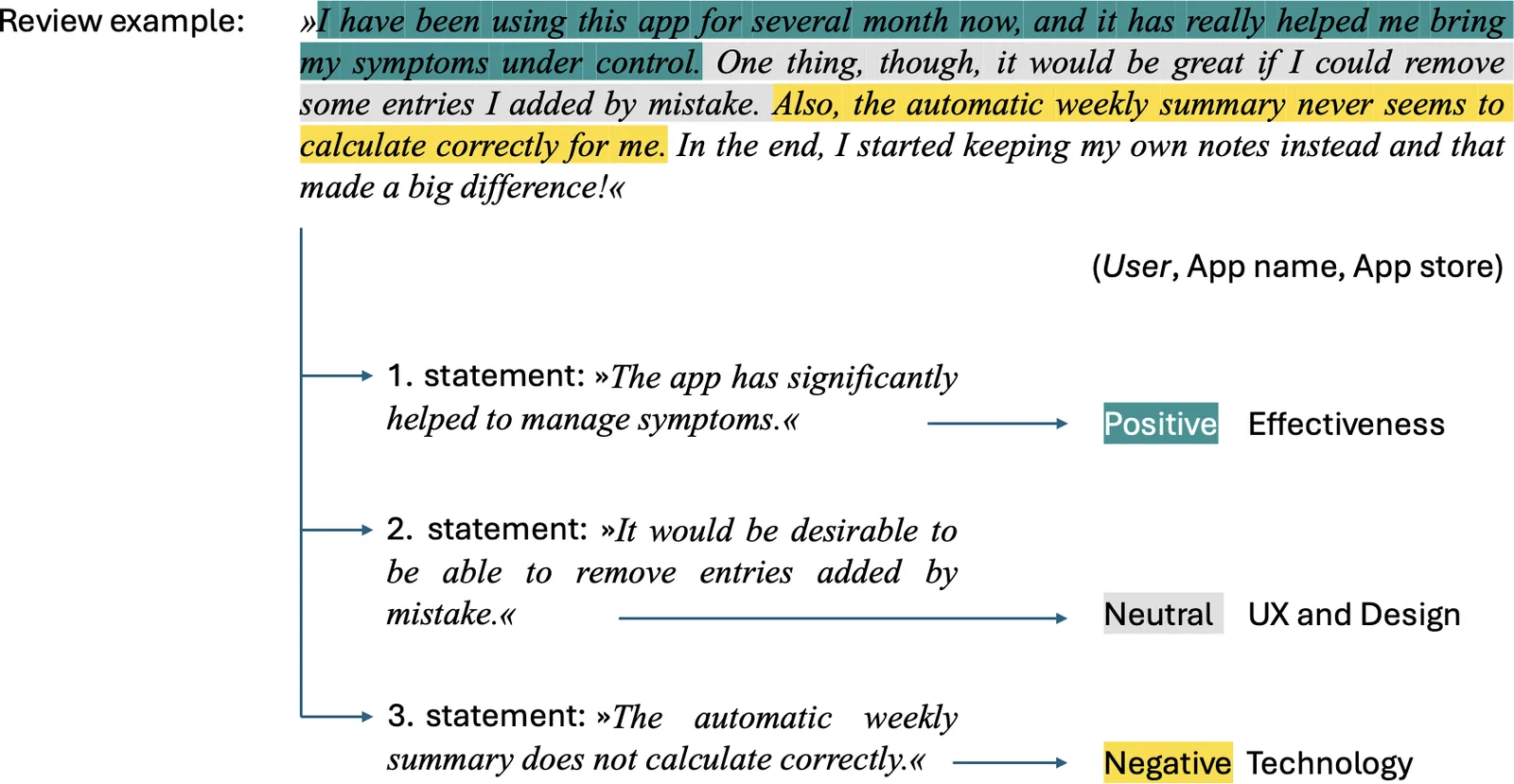
Illustrative example of review classification process. UX: user experience.

### Human Validation of Model Output

The accuracy and conceptual validity of the model-generated classifications were evaluated through a structured validation process, as shown in Figure 2. The full set of reviews, including the original review, extracted statement, sentiment label, and categories, was evenly divided among 3 authors (step 7), each of whom independently reviewed one-third of the dataset (step 8). The validation process followed a four-step protocol given below:

1. Independent review: Each model-generated statement and its assigned sentiment and category were assessed in relation to the original review. Reviewers evaluated whether the extracted statement captured a meaningful aspect of the review and whether the sentiment and category were accurate according to the predefined classification framework.
2. Flagging of inconsistencies: Statements that were incomplete, overly generic, redundant, or incorrectly classified (eg, in sentiment or category) were flagged. Each flagged item included a brief comment and a suggested correction, if appropriate.
3. Triangulated reassessment: The 2 remaining authors independently evaluated the flagged items, providing their own classifications without being influenced by the previous assessments (step 9). This resulted in 3 independent judgments per flagged entry.
4. Consensus resolution: In cases of disagreement, the authors discussed each item until a final classification was agreed upon (step 10).

**Figure 2.**
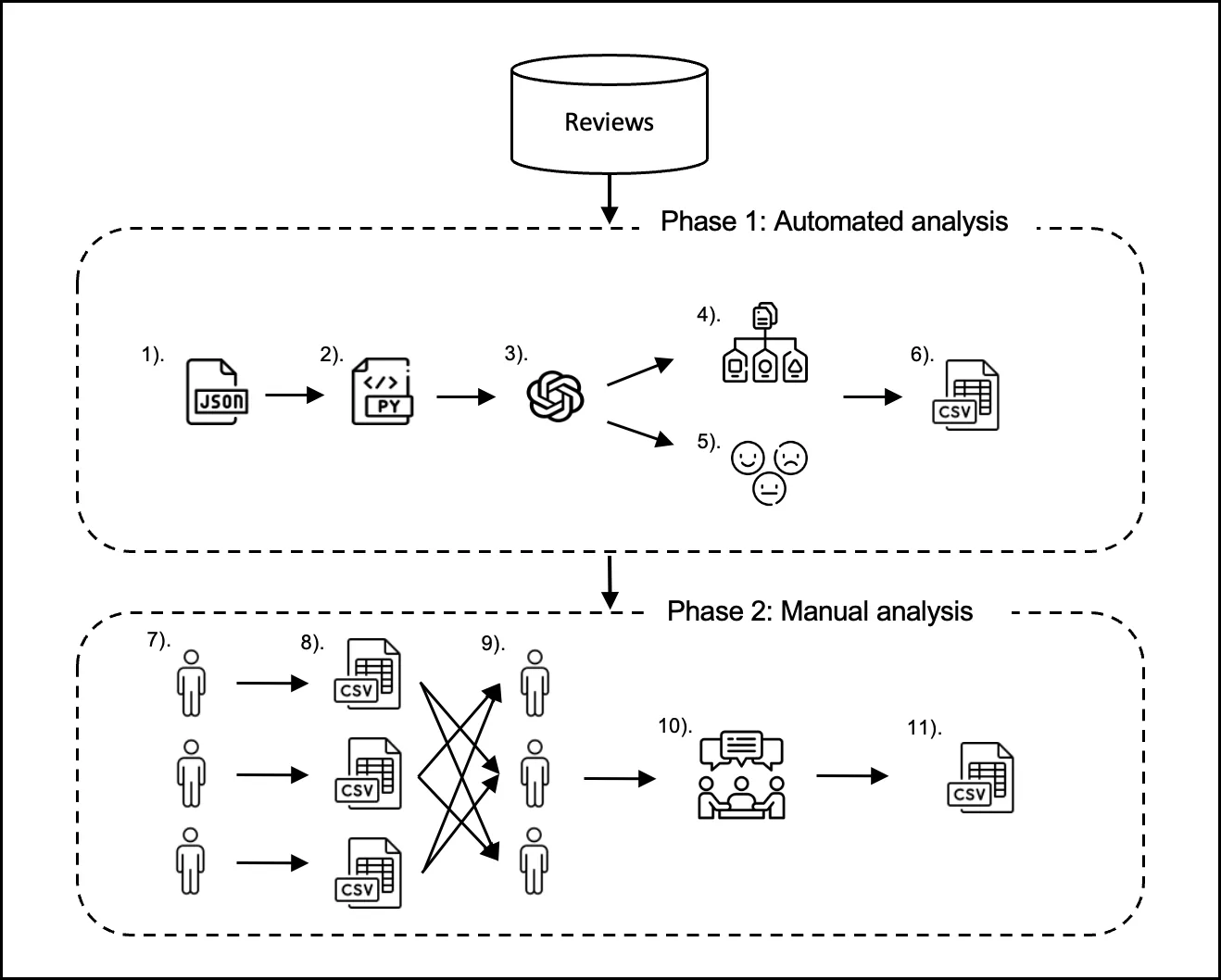
Overview of the 2-phase classification and sentiment analysis process, combining automated extraction and classification (phase 1) with manual validation and consensus (phase 2).

Validated statements were finalized and included in the analysis as a consolidated CSV dataset (step 11). Structurally flawed outputs (eg, empty responses or parsing errors) were reprocessed. The manual validation ensured consistency with the classification framework and enabled direct evaluation of the model on real-world data.

### Data Analysis

Following the classification and validation process, a descriptive analysis was conducted. The proportions of positive, neutral, and negative statements were calculated for the full dataset and for each of the 10 categories. Data processing and descriptive analyses were performed in Python using the *pandas* library (version 2.2.3), while figures were generated using *matplotlib* (version 1.3.0). In addition, the validated statements within each category were qualitatively reviewed by the authors to identify recurring content patterns among the positive, neutral, and negative statements. The category-specific findings reported in the “Results” section, including descriptions of frequently mentioned issues, are based on this qualitative review. Because a single app accounted for a disproportionately high share of the dataset, a sensitivity analysis was performed in which all analyses were repeated after excluding this app to assess whether its disproportionate weight biased the overall results. In addition, because statements originating from the same app are unlikely to be fully independent, all analyses were additionally repeated using an app-level averaging approach. In this clustering-adjusted analysis, sentiment proportions were calculated separately for each app and then averaged across apps so that every app contributed equally regardless of the number of statements it provided.

Beyond the predefined statement-level classification, an exploratory BERTopic analysis was conducted on all included reviews using a multilingual sentence transformer model (paraphrase-multilingual-mpnet-base-v2) to assess whether unsupervised topic modeling could provide a suitable approach [24]. Topics were inspected qualitatively based on their top terms and compared with the 10 predefined categories. Based on this assessment, BERTopic was not used for the final quantitative analysis. The full topic modeling output can be found in Multimedia Appendix 3.

### Ethical Considerations

This study analyzed publicly accessible user reviews voluntarily submitted to the German Apple App Store and the Google Play Store. The study involved no interaction with review authors, no recruitment of participants, and no access to clinical records, nonpublic personal data, or other restricted information. The analysis was observational and based exclusively on publicly available product evaluations. Following the context-sensitive framework proposed by Eysenbach and Till [25], we considered not only the public accessibility of the reviews but also the nature of the online setting, the noninteractive study design, and potential risks to review authors. App store reviews were treated as publicly accessible, one-directional product evaluations rather than communications within a bounded virtual community. The reviews could be viewed without registration and did not involve an expectation of interpersonal exchange or membership in a restricted group. The analysis of publicly available app store reviews is an established approach in mHealth research and has been used to investigate user experience, usability, perceived benefits, and recurring barriers to app use [13,20-22]. To reduce potential privacy risks, analyses were conducted and reported primarily in aggregate form. No usernames, account identifiers, or other direct identifiers were included in the analytical dataset or reported in the manuscript. Because verbatim quotations may enable reidentification through search engines, no direct quotations from individual reviews are reported in this manuscript. Formal ethics review was not sought because the study involved a noninteractive analysis of publicly accessible app store reviews and did not involve the collection of nonpublic personal data.

## Results

### Overview of the Dataset

As of February 25, 2025, a total of 69 DiGAs were listed in the official directory maintained by the German BfArM. Of these, 45 DiGAs were available as mobile apps (iOS and Android), and 34 offered a web-based version, with several products available in both formats. Since DiGA-user reviews are only available for mobile apps distributed through public app stores, the analysis was limited to the mobile app versions of DiGAs. Web-based applications were excluded due to the lack of publicly accessible user feedback.

Although 45 DiGAs had a mobile app, 1 DiGA had no publicly available user reviews and could therefore not be analyzed. Thus, 44 DiGAs were included in the analysis. Among them, 29 (65.9%) had been permanently approved, and 15 (34.1%) were listed on a provisional basis at the time of data collection. It is important to note that several DTx share a common mobile app. For example, the *HelloBetter* app includes 6 distinct DiGAs addressing different indications (eg, chronic pain, diabetes, sleep disorders, panic, stress, and vaginismus), all delivered through the same app infrastructure. Similarly, the *Selfapy* app delivers 5 approved DiGAs (eg, for depression, bulimia nervosa, generalized anxiety disorder, and chronic pain) through a single mobile app. Accordingly, the 44 DiGAs available as mobile apps are represented by 35 mobile apps. Consequently, the app-based analysis cannot fully differentiate between user feedback for each individual DiGA when multiple products are bundled within one app. Nevertheless, all reviews associated with such apps were included in the analysis, as they reflect real-world user experiences with the respective DiGA ecosystem.

From these apps, 4410 publicly accessible user reviews were extracted via the browser-based versions of the German Apple App Store and the German Google Play Store. For the Apple App Store, only up to 10 written reviews are publicly visible per app when accessed via a browser. During data cleaning and preprocessing, 30 reviews could not be processed further due to incomplete content, parsing errors, or other technical parsing errors. These entries were excluded from the analysis. The remaining dataset of 4380 reviews was fully included in the quantitative and sentiment-based evaluation.

### Validation of Model Output

From the 4380 included user reviews, a total of 9494 individual statements were extracted and systematically categorized. Each statement was assigned both a sentiment label (positive, neutral, or negative) and a thematic category. The initial classification was conducted by an LLM, followed by the manual validation. During this manual validation, 28 reviews were excluded due to irrelevance or ambiguous content. These reviews primarily included off-topic comments, incomplete sentences, and noninformative content unrelated to the DTx itself—such as emoji-only responses, promotional slogans, or vague remarks like “I want to be a hero. Greatings, Josef, *“*just installed*,”* or “don’t know yet.” In addition, 27 statements (corresponding to 24 reviews) were flagged as technically erroneous and removed. Statements addressing aspects outside the 10 predefined categories were rarely observed during validation. Together, manual-validation exclusions (28 reviews) and the technical exclusions (24 reviews) affected a total of 52 user reviews that were not included in the final dataset. As a result, the final dataset comprises 4328 user reviews containing at least one interpretable statement and a total of 9439 valid and interpretable statements. The complete data processing workflow, including all extraction, classification, and exclusion steps, is summarized in Figure 3. The complete classified dataset is provided in Multimedia Appendix 4. These 9439 statements form the basis for all subsequent quantitative analyses presented in this study. On average, a review consisted of 213.9 characters and 2.2 relevant statements. A detailed overview of the distribution of valid reviews and statements across individual apps can be found in Multimedia Appendix 4*.*

**Figure 3.**
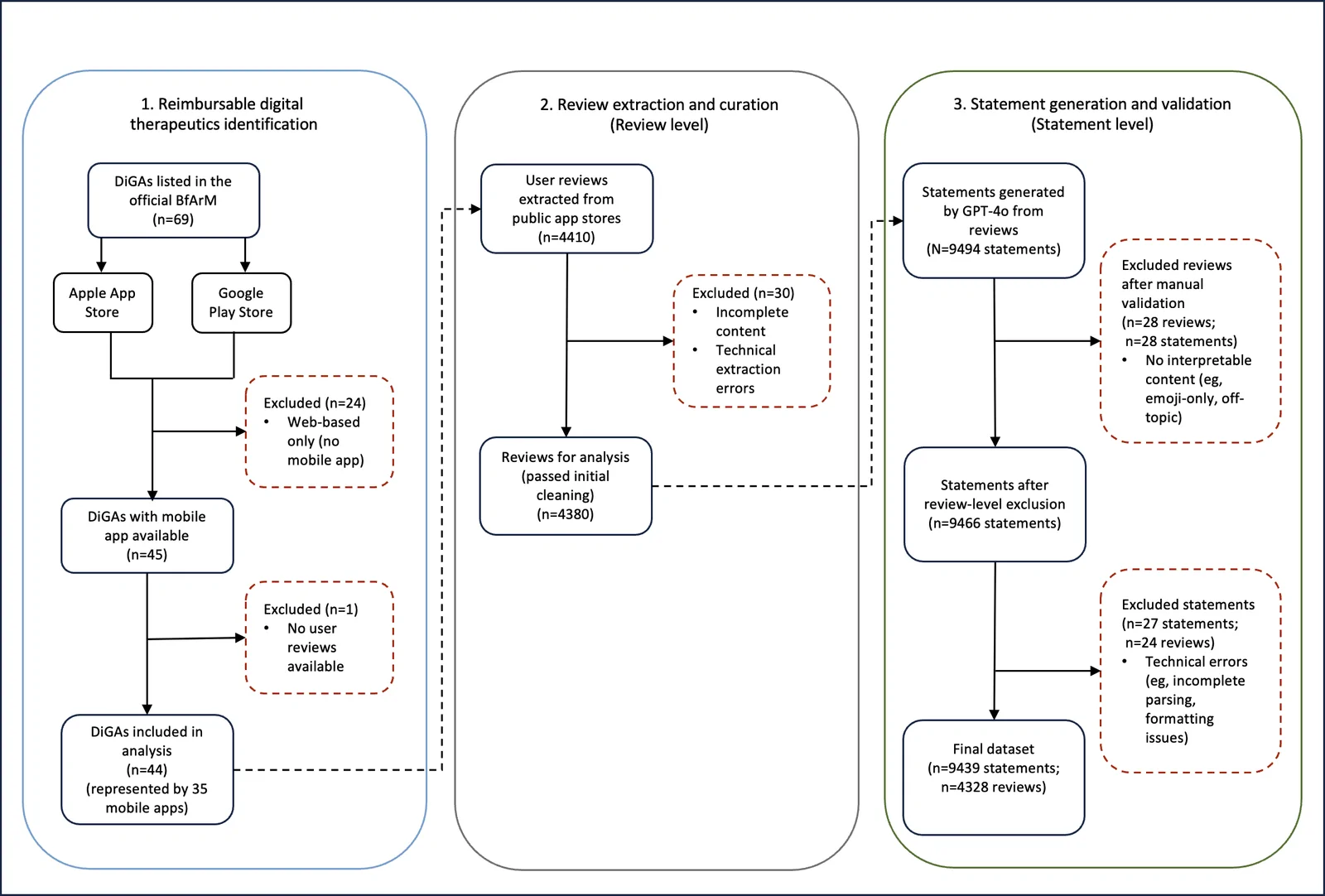
Workflow of review data identification, extraction, cleaning, and validation. DiGa: digital health application.

### Agreement Between Initial Model Output and Human-Validated Classification

To assess the reliability of the automated sentiment and category classification, a systematic manual validation was performed. Agreement between the initial GPT-4o classifications and the final human-validated classifications was high. For sentiment labels, the overall agreement was 99%, with excellent precision and recall for positive (n=6491; *F*_1_ score=1.00) and negative statements (n=2474; *F*_1_-score=0.99) and for neutral statements (n=474; *F*_1_-score=o.92). The sentiment confusion matrix (Figure 4a) shows minimal misclassifications, primarily between neutral and negative labels.

For category classification, agreement between the initial model output and the final human-validated classifications was 95%, with an average *F*_1_-score of 0.95 (Figure 4b). Most thematic agreements were consistent with human reviewers’ assessments, particularly for frequently occurring categories such as *overall impression* (n=1431; *F*_1_-score=0.96), *content* (n=2564; *F*_1_-score=0.95), *effectiveness* (n=1609; *F*_1_-score=0.94), and *support* (n=865; *F*_1_-score=0.98). However, lower agreement rates were observed for categories with fewer labeled examples, such as *prescription/approval* (n=234; *F*_1_-score=0.89) and *login/registration* (n=280; *F*_1_-score=0.92).

**Figure 4.**
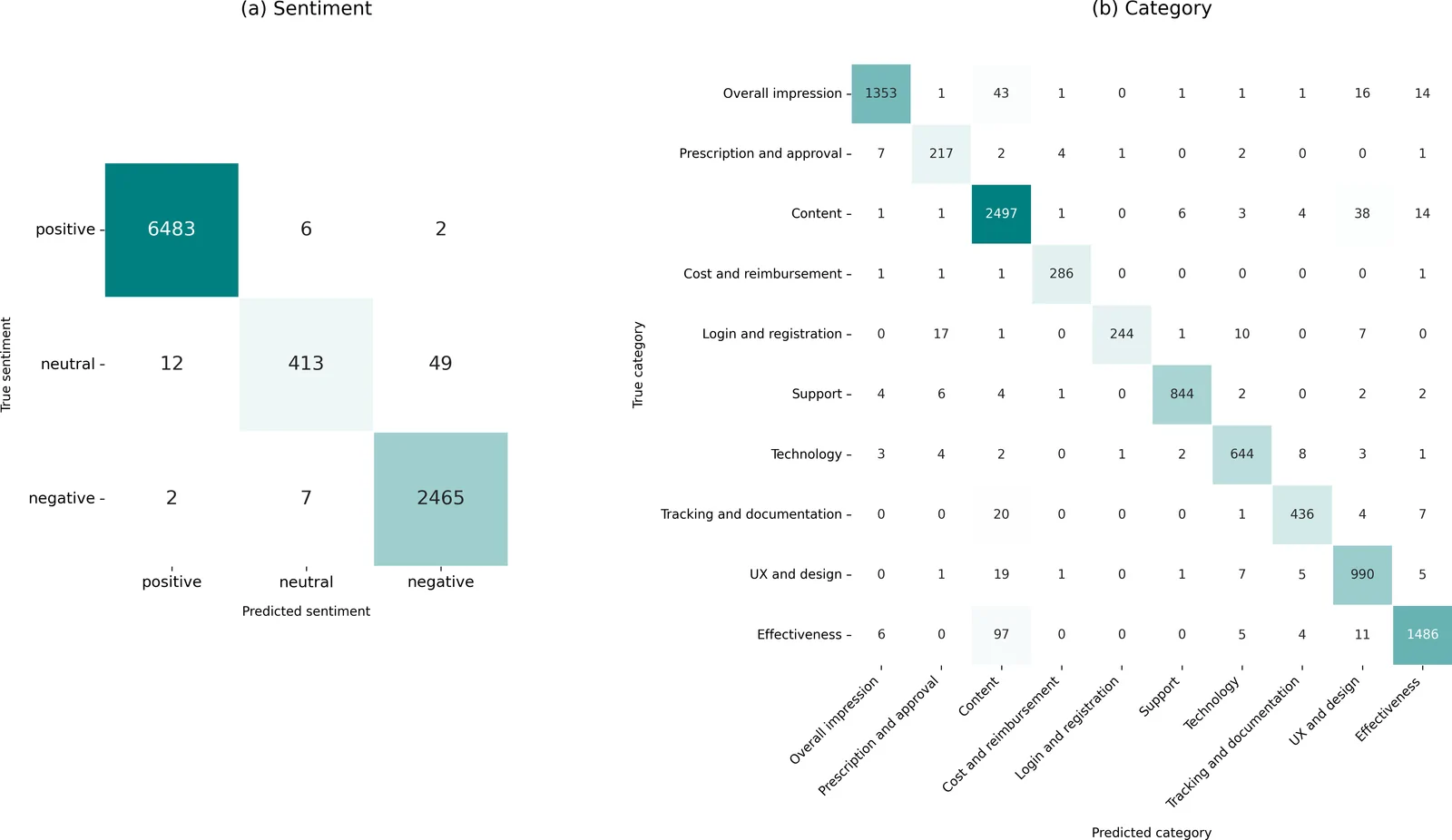
Confusion matrices for the model-based classification: (a) sentiment and (b) category.

### Distribution of Sentiments and Categories

In total, 6491 out of 9439 (68.8) statements were classified as positive, 474 out of 9439 (5.0%) statements as neutral, and 2474 out of 9439 (26.2%) statements as negative. The majority of positive statements were assigned to the category *content* (n=2001, 30.8%), followed by *effectiveness* (n=1440, 22.2%) and *overall impression* (n=1233, 19.0%). Among the 474 neutral statements, most were related to *content* (n=145, 30.6%), *UX/design* (n=83, 17.5%), and *effectiveness* (n=68, 14.3%). Negative classifications (n=2474) occurred most frequently in the categories *Technology* (n=563, 22.8%), *UX/design* (n=340, 13.7%), and *login/registration* (n=263, 10.6%) (Figure 5a ). A sensitivity analysis excluding the DTx Zanadio (Sidekick Health Germany GmbH), which accounted for 1433 of 4328 (33.1%) reviews, showed a slightly lower proportion of positive statements (65% vs 68.8% in the full dataset) but did not change the overall pattern of the results, with the same categories dominating positive and negative feedback (Figure 5b).

**Figure 5.**
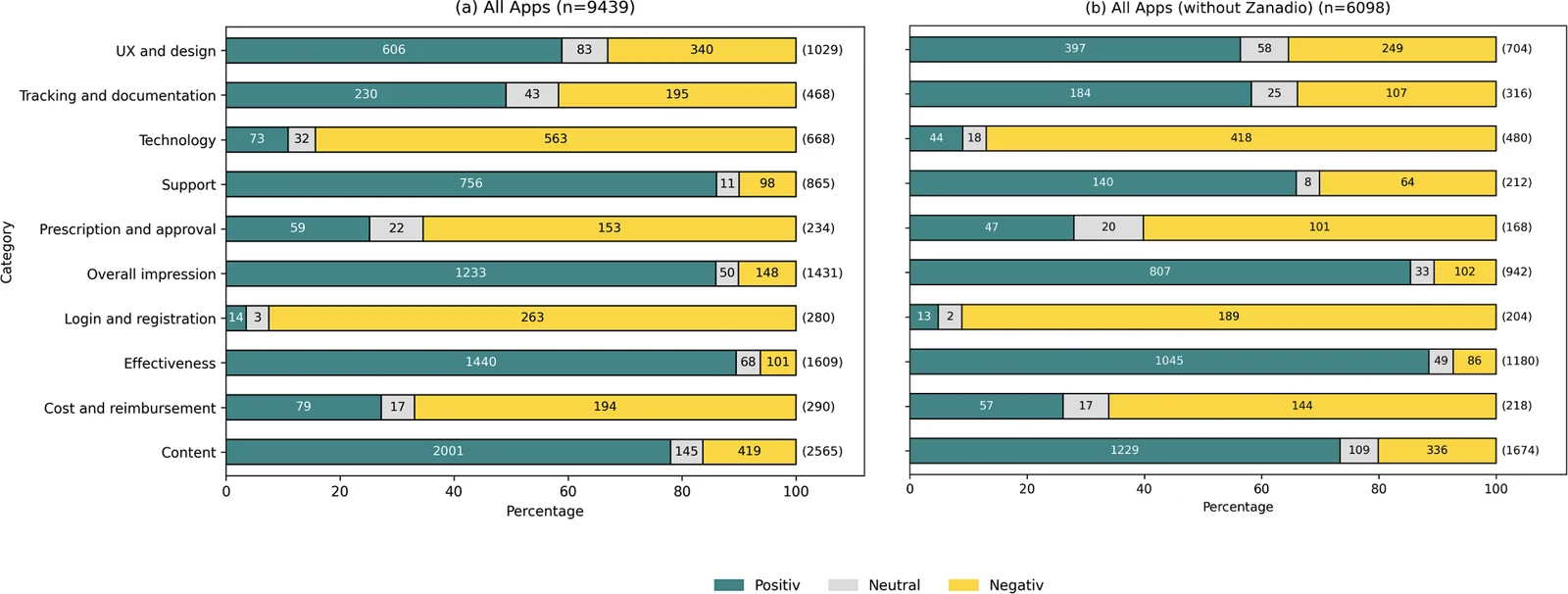
Sentiment distribution per category across all apps (a) and excluding Zanadio (b).

A clustering-adjusted analysis, in which sentiment proportions were averaged across apps rather than pooled across statements, showed comparable results (64.2% vs 65.0% [3963/6098] positive statements when excluding the most frequently reviewed app) and did not change the ranking of the most positively and most negatively evaluated categories. Larger deviations occurred only in the categories cost and reimbursement and support, in which the median number of statements per app was below 5, making estimates more sensitive to individual reviews. The full comparison is provided in Multimedia Appendix 5.

In addition to the aggregated results, sentiment distributions were also analyzed separately for each individual DTx included in the dataset. The detailed graphical breakdown of sentiment and thematic categories per app is provided in Multimedia Appendix 6 for reference. This allows a more granular exploration of potential differences in user feedback across specific DTx products, beyond the aggregated trends presented in the “Result” section.

### Category-Specific Findings

The following sections provide a detailed overview of user feedback for 3 selected categories: *overall impression*, *effectiveness*, and *login/registration*. These categories were chosen due to their high frequency and relevance across the dataset. Analyses of additional categories, such as *UX/design*, *tracking/documentation*, *technology*, *support*, *prescription/approval*, *cost/reimbursement*, and *content* are provided in Multimedia Appendix 1 for reference.

#### Overall Impression

A total of 1431 user statements were categorized under “overall impression”, providing general user feedback regarding the DTx experience. Positive reviews frequently emphasized the usefulness, clarity, and motivational aspects of the apps. Several users described individual programs as motivating, adaptable to personal needs, and worth recommending. Others highlighted that an app helped them understand and manage specific health concerns and praised its intuitive, clearly structured design, or noted that exploring the relationship between their condition and an everyday factor was insightful and that the app was easy to use. Neutral sentiments reflected initial or uncertain user experiences, with some users indicating only a tentative first impression or that they were not yet convinced. Reviews with negative statements often criticized technical problems or unmet expectations, such as poor performance and a lack of personalization due to a nonadjustable algorithm, or noted that, while an app supported the documentation of symptoms, it did not substitute for therapy and provided no personal benefit.

#### Login/Registration

In total, 280 user statements addressed the login and registration process. Positive feedback frequently highlighted an easy onboarding experience and simple account handling, with users describing straightforward registration, clear profile setup, and quick login as features that made it easy to get started. Neutral reviews often paired a generally positive impression with specific suggestions for improvement, such as the wish to have their login data or the email address saved to reduce repeated entry. Negative experiences primarily concerned technical login problems or authentication procedures perceived as overly complex. Some users reported losing access to their account after an update and being frustrated by having to log in repeatedly, while others described persistent login failures independent of their email provider. Several users specifically objected to a 2-step login process, which they considered unnecessary for this type of app.

#### Effectiveness

The category “effectiveness” received 1609 user statements, making it one of the most frequently mentioned aspects of the DTx evaluations. Many users reported positive effects on their health, highlighting improved well-being, symptom relief, or successful integration of the app into daily routines. Users described being satisfied with the range of features and reported that an app had genuinely helped them or that a sleep-focused app had improved how quickly they fell asleep and how well they stayed asleep, while praising its clear structure. Neutral reviews often reflected mixed impressions or external limitations affecting the expected effectiveness, such as finding reminders simultaneously bothersome and necessary or struggling with personal motivation despite valuing the content. Others characterized an app as a helpful first step toward understanding their condition, one that nonetheless required discipline and could not replace professional medical care. Reviews with negative statements often expressed disappointment about insufficient health improvements, with some users noting that helpful tips ultimately did not work for them. A more critical assessment reported no improvement despite full completion of the program, criticized the high cost, reimbursed by health insurance, relative to the benefits, and described the app as poorly designed and unstable.

## Discussion

### Principal Results

This study presents a large-scale analysis of publicly available app store reviews for reimbursable DiGAs in Germany, based on 9439 classified user statements across 44 listed DTx. Our results show that user feedback was predominantly positive with 6491 of 9439 (68.8%) classified as positive. Positive sentiment was particularly concentrated in the categories effectiveness (1440/1609, 89.5% positive), overall impression (1233/1431, 86.2% positive), and content (2001/2565, 78.0% positive), indicating that users who submitted reviews frequently expressed favorable views of the therapeutic approach and content of these apps. At the same time, 2474 of 9439 (26.2%) statements were negative, with login and registration, technology, and prescription and approval emerging as the most frequently criticized dimensions, pointing to persistent barriers in access, technical reliability, and administrative processes. The high overall classification performance of the model (99% sentiment accuracy, 95% category accuracy) supports the reliability of these patterns.

The predominantly positive overall sentiment, particularly in the categories of effectiveness and content, suggests that many users may perceive DiGAs as therapeutically meaningful and that the content quality of many apps may meet user expectations. This finding should be interpreted in light of known review behavior, as app store reviews tend to be predominantly positive overall, with praise being the most frequently expressed topic [26]. The high proportion of positive statements regarding effectiveness is therefore best understood as an indicator of perceived, self-reported benefit rather than a validated therapeutic effect. Notably, perceived effectiveness from the user perspective has also gained regulatory attention, as the app-accompanying assessment under section 139e (13) SGB V has included patient-reported health status during the DiGA use since January 1, 2026 [3].

In contrast, the high share of negative statements in the categories technology, login and registration, and prescription and approval points to structural barriers that undermine user access and sustained engagement. Technical malfunctions and app instability are particularly concerning given that DiGAs are regulated medical devices subject to quality and safety requirements under section 139e Abs 2 SGB V. Frequent user criticism of bugs and crashes suggests a gap between regulatory certification and real-world software quality, indicating a need for improved release and quality management by manufacturers [27]. From a requirements engineering perspective, technical stability and reliable access represent basic requirements or “must-be” factors whose absence reliably generates negative feedback, regardless of, for example, an app’s therapeutic value [28].

The criticism directed at login and registration processes reflects a broader tension between security requirements and usability. Multifactor authentication, while aligned with national cybersecurity standards, appears to conflict with the low-threshold access that digital regulations expect for a user population with an average age of 55-60 years [6]. Similarly, the prescription and approval category reveals user frustration with the administrative burden of the reimbursement pathway, including lengthy insurer approval processes, eligibility restrictions, and the recurring 90-day prescription cycle. Some users additionally expressed a desire to trial apps before committing to a prescription, which aligns with broader arguments in the literature for more flexible access models in DTx [12]. This concern is in line with broader critiques of the DiGA framework, in which the activation and prescription process and the lack of trial options have been identified as barriers to acceptance among prescribing physicians [11].

Compared to Uncovska et al [19], who applied unsupervised BERTopic modeling to a mixed sample of regulated and nonregulated mHealth apps, our study focuses exclusively on officially listed DiGAs and maps user feedback to a predefined framework grounded in regulatory criteria. Rather than discovering latent topics, our approach systematically evaluates user experience along dimensions that are directly relevant to DiGA approval and postmarket monitoring. This regulatory alignment allows for more targeted interpretation of findings and facilitates direct translation into policy and quality improvement recommendations. However, such reviews cannot replace formal postmarket surveillance or systematically collected patient-reported outcome measures, as they are subject to selection bias and may not represent the broader user population [8,9].

### Limitations

Several methodological limitations should be considered when interpreting these findings. App store reviews are voluntary and may be affected by self-selection bias, as users with particularly positive or negative experiences may be more likely to provide feedback, whereas neutral experiences may be underrepresented [26]. The reviews are submitted without a standardized clinical or usage context. Thus, they may reflect isolated incidents rather than sustained use, and cannot be considered representative of all DiGA users. In addition, app store reviews are not a curated or audited source, and prior research has identified potentially incentivized or inauthentic reviews in app stores [29]. No additional bot detection or fraud screening beyond the app stores’ own moderation was applied, and neither users’ identity nor patient status could be verified. They should therefore be interpreted as complementary insights into perceived user experience and usability rather than as a replacement for formal clinical evaluation or postmarket surveillance. The validation process was not based on a fully independent blinded double-coding design. The 3 reviewers assessed and corrected model-generated statements and labels in relation to the original review rather than independently annotating all reviews from scratch. Although this enabled the validation of a large dataset, it may have introduced anchoring or acceptance effects. The reported agreement measures therefore reflect concordance between the initial model output and the final human-validated dataset, rather than an independent estimate of model accuracy or interrater reliability. The predefined category framework, while based on legal, regulatory, and quality-related criteria for reimbursable DiGAs, may not fully capture user concerns outside these dimensions, such as social support, gamification, emotional well-being, or motivational factors. As the framework was derived from regulatory requirements, it captures the quality dimensions that are legally relevant for DiGAs, but not necessarily features that go beyond these requirements and may generate particular enthusiasm among users. Identifying the latter would require an inductive coding approach, which presents a promising direction for future research. In addition, reviews were available at the level of mobile apps rather than individual DiGAs. Because several apps bundled multiple DiGAs, feedback could not always be assigned reliably to a specific product, indication, or listing status. Moreover, a single app accounted for approximately one-third of all reviews, so the aggregated findings are weighted toward the most frequently reviewed apps. A sensitivity analysis excluding this app confirmed the overall sentiment distribution and category patterns, but the pooled percentages should be interpreted with this imbalance in mind.

Finally, the analysis did not investigate the temporal development of reviews in detail. Future longitudinal analyses could assess whether technology-related complaints were transient, for example following app launches or updates, or reflected persistent barriers [30].

### Comparison With Prior Work

Our findings are consistent with prior app review research showing that technical issues, registration barriers, and usability problems are recurring concerns in mHealth apps [14,19]. However, by linking user feedback to regulation-informed categories, our study adds a different perspective: it shows which aspects of the DiGA approval framework are reflected in users’ real-world experience and where practical barriers persist despite regulatory certification. In this sense, the study extends prior exploratory work by demonstrating how app store reviews can be used not only to identify general user concerns, but also to generate user-reported real-world evidence relevant to postmarket monitoring and regulatory quality improvement. Similar patterns emerge in other contexts. In an analysis of app store reviews for prescription DTx cleared by the US Food and Drug Administration, access barriers, technical problems, and engagement limitations were among the most frequent themes [20]. A thematic analysis of 13,549 reviews of mental health apps identified poor usability as the most common reason for app abandonment, along with registration requirements perceived as excessive [21]. Overall sentiment in the US sample was predominantly negative, in contrast to our largely positive findings, which may reflect differences in reimbursement or prescription pathways.

### Conclusions

This study provides a large-scale overview of how users experience and evaluate reimbursable DTx in Germany, based on publicly available app store reviews. The findings show that publicly available app store reviews can reveal practical barriers that affect whether reimbursable DTx are accessible, usable, and acceptable in everyday care. Although user feedback was predominantly positive, especially regarding perceived effectiveness, overall impression, and content, recurring criticism of login and registration processes, technical reliability, and prescription and approval procedures indicates that users continue to encounter barriers at key points of access and use.

The practical relevance of these findings lies in the fact that many of the criticized aspects are not peripheral convenience issues, but basic prerequisites for sustained engagement with medical-grade digital health apps. Technical stability, reliable access, understandable onboarding, and low-threshold registration should therefore be treated as essential implementation requirements. App store reviews may help regulators, manufacturers, and researchers identify such barriers early and complement formal evaluation procedures with user-centered real-world feedback. LLM-assisted approaches can support this process by enabling large volumes of unstructured user feedback to be analyzed in a structured and scalable way. As the DiGA framework matures, systematically incorporating user-reported real-world evidence into postmarket surveillance could help ensure that regulatory certification translates into DTx that are not only proven to be effective in clinical trials but also accessible and usable in everyday care.

## Supplementary material

10.2196/92415 Multimedia Appendix 1 Legal basis and results for user feedback categories in DiGA app store reviews.

10.2196/92415 Multimedia Appendix 2 LLM-Prompt.

10.2196/92415 Multimedia Appendix 3 BERTopic modeling output.

10.2196/92415 Multimedia Appendix 4 App store user reviews.

10.2196/92415 Multimedia Appendix 5 Clustering-adjusted analysis.

10.2196/92415 Multimedia Appendix 6 Category and sentiment analysis per App.

## Abbreviations

BfArM: Bundesamt für Arzneimittel und Medizinprodukte
DiGA: digital health application
DiGAV: Digital Health Application Regulation
DTx: digital therapeutics
LLM: large language model
mHealth: mobile health
SGB V: German Social Code, Book V
UX: user experience
