# Supplementary material for: What Users Say About Reimbursable Digital Therapeutics in Germany: Large-Scale App Store Review Analysis Using a Large Language Model
Source: JMIR Hum Factors. 2026 Oct 8;13:e92415. doi: 10.2196/92415 (PMC13649173; doi:10.2196/92415)
Supplement: Supplementary file 1 [file humanfactors-v13-e92415-s001.pdf]

Multimedia Appendix 1:

Methods:

Legal and Regulatory References for User Feedback Categories in DTx/ DiGA Evaluation

**Category**	**Associated DiGA Criteria**	**Legal Basis (Original German Reference)**	**Legal Basis (English Reference Version)** *Translated from the original German using ChatGPT-5*
Content	Content quality, evidence base, up-to-dateness, scientific soundness, readability	§ 139e Abs. 2 SGB V: "Voraussetzung für die Aufnahme in das Verzeichnis [...] ist, dass [...] die digitale Gesundheitsanwendung [...] den Anforderungen an Sicherheit, Funktionstauglichkeit, Qualität, Datenschutz und Datensicherheit entspricht."<br><br>DiGAV § 5 Abs. 8 Satz 2: "Sofern die digitale Gesundheitsanwendung die Versicherten mit Gesundheitsinformationen unterstützt, müssen die Gesundheitsinformationen ebenfalls dem allgemein anerkannten fachlichen Standard entsprechen und zielgruppengerecht aufbereitet sein."<br><br>DiGAV § 5 Abs. 8: "(8) Die von digitalen Gesundheitsanwendungen verwendeten medizinischen Inhalte müssen dem allgemein anerkannten Stand der medizinischen Erkenntnisse entsprechen. Sofern die digitale Gesundheitsanwendung die Versicherten mit Gesundheitsinformationen unterstützt, müssen die Gesundheitsinformationen ebenfalls dem allgemein anerkannten fachlichen Standard entsprechen und zielgruppengerecht aufbereitet sein."	§ 139e (2) SGB V: "A prerequisite for inclusion in the directory [...] is that [...] the digital health application [...] complies with the requirements for safety, functional capability, quality, data protection, and data security."<br><br>§ 5 (8) sentence 2 DiGAV: "If the digital health application provides insured persons with health information, this information must also comply with generally accepted professional standards and be prepared in a way that is appropriate for the target group."<br><br>§ 5 (8) DiGAV: "(8) The medical content used by digital health applications must comply with the generally accepted state of medical knowledge. If the digital health application provides insured persons with health information, this information must also comply with generally accepted professional standards and be prepared in a way that is appropriate for the target group."
Technology	Technical stability, system compatibility, update frequency, bug handling	§ 139e Abs. 2 SGB V: "Voraussetzung für die Aufnahme in das Verzeichnis [...] ist, dass [...] die digitale Gesundheitsanwendung [...] den Anforderungen an	§ 139e (2) SGB V: "A prerequisite for inclusion in the directory [...] is that [...] the digital health application [...] meets the requirements for safety, functionality, quality, data protection, and data security."

		Sicherheit, Funktionstauglichkeit, Qualität, Datenschutz und Datensicherheit entspricht."	
		DiGAV §5 Abs. 2: "(2) Digitale Gesundheitsanwendungen sind so zu gestalten, dass sie robust gegen Störungen und Fehlbedienungen sind."	§ 5 (2) DiGAV: "(2) Digital health applications must be designed to be robust against malfunctions and incorrect use."
		Leitfaden BfArM (2023), Abschnitt 3.3.2: "Technische Stabilität, Kompatibilität mit Endgeräten und regelmäßige Updates sind sicherzustellen."	BfArM Guideline (2023), Section 3.3.2: "Technical stability, compatibility with end devices, and regular updates must be ensured."
Cost/Reimbursement	Perceived cost, issues with reimbursement by health insurance	§ 134 Abs. 1 SGB V: "Die Vergütung erfolgt [...] auf der Grundlage einer Vereinbarung mit dem GKV-Spitzenverband."	§ 134 (1) SGB V: "Remuneration shall be based [...] on an agreement with the National Association of Statutory Health Insurance Funds (GKV-Spitzenverband)."
Login/Registration	Initial access, ease of login or registration	DiGAV § 5 Abs. 5: "(5) Digitale Gesundheitsanwendungen sind so zu gestalten, dass die Versicherten diese leicht und intuitiv bedienen können."	§ 5 (5) DiGAV: "(5) Digital health applications must be designed in such a way that insured persons can use them easily and intuitively."
Prescription/Approval	Problems with prescription, approval by health insurance, diagnosis requirements	Leitfaden BfArM (2023), Abschnitt 3.2.2: "Der Zugang zu DiGAs über Krankenkassen und digitale Rezepte muss gewährleistet sein."	BfArM Guideline (2023), Section 3.2.2: "Access to DiGAs via health insurance funds and digital prescriptions must be ensured."
		Leitfaden BfArM (2023), Abschnitt 2.1.2.2: "Versicherte können sich auch ohne ärztliche Verordnung eine gelistete DiGA durch die Krankenkasse genehmigen lassen.“	BfArM Guideline (2023), Section 2.1.2.2: "Insured persons may also obtain approval for a listed DiGA from their health insurance fund without a medical prescription."
UX/Design	User interface, usability, visual and design aspects	DiGAV § 5 Abs. 5: "(5) Digitale Gesundheitsanwendungen sind so zu gestalten, dass die Versicherten diese leicht und intuitiv bedienen können."	§ 5 (5) DiGAV: "(5) Digital health applications must be designed in such a way that insured persons can use them easily and intuitively."
Support	Availability, friendliness, and quality of customer support	Leitfaden BfArM (2023), Abschnitt 3.6.2.5: „Die DiGAV fordert, dass der Hersteller innerhalb von 24 Stunden auf Anfragen reagiert.“	BfArM Guideline (2023), Section 3.6.2.5: "The DiGAV requires that the manufacturer responds to inquiries within 24 hours."
Tracking/Documentation	Documentation of symptoms, nutrition, activities, or behaviors	* § 139e Abs. 13 SGB V: "Zu den Ergebnissen der anwendungsbegleitenden Erfolgsmessung nach Satz 1 zählen insbesondere [...] der patientenberichtete	* § 139e (13) SGB V: "The results of the accompanying evaluation of effectiveness pursuant to sentence 1 shall

		Gesundheitszustand während der Nutzung der digitalen Gesundheitsanwendung."<br><br>** Will become effective on January 1, 2026.*	include, in particular, the patient-reported health status during the use of the digital health application."<br><br>** Will become effective on January 1, 2026.*
Effectiveness	Perceived medical benefit, self-management, health literacy, therapy adherence	§ 139e Abs. 13 SGB V: "Zu den Ergebnissen der anwendungsbegleitenden Erfolgsmessung nach Satz 1 zählen insbesondere [...] der patientenberichtete Gesundheitszustand während der Nutzung der digitalen Gesundheitsanwendung."<br><br>DiGAV § 8 Abs. 2: "Der medizinische Nutzen im Sinne dieser Verordnung ist der patientenrelevante Effekt insbesondere hinsichtlich der Verbesserung des Gesundheitszustands, der Verkürzung der Krankheitsdauer, der Verlängerung des Überlebens oder einer Verbesserung der Lebensqualität."<br><br>DiGAV § 8 Abs.3: "(3) Die patientenrelevanten Struktur- und Verfahrensverbesserungen in der Versorgung im Sinne dieser Verordnung sind im Rahmen der Erkennung, Überwachung, Behandlung oder Linderung von Krankheiten oder der Erkennung, Behandlung, Linderung oder Kompensierung von Verletzungen oder Behinderungen auf eine Unterstützung des Gesundheitshandelns der Patientinnen und Patienten oder eine Integration der Abläufe zwischen Patientinnen und Patienten und Leistungserbringern ausgerichtet und umfassen insbesondere die Bereiche der [...] 2. Ausrichtung der Behandlung an Leitlinien und anerkannten Standards, 3. Adhärenz, [...] 6. Gesundheitskompetenz, 7. Patientensouveränität, 8.Bewältigung krankheitsbedingter Schwierigkeiten im Alltag, [...]"	§ 139e (13) SGB V: "The results of the accompanying evaluation of effectiveness pursuant to sentence 1 shall include, in particular, the patient-reported health status during the use of the digital health application."<br><br>DiGAV § 8 (2):<br>"The medical benefit within the meaning of this regulation refers to the patient-relevant effect, particularly with regard to improving health status, shortening the duration of illness, prolonging survival, or improving quality of life."<br><br>DiGAV § 8 (3): "(3) The patient-relevant structural and procedural improvements in care within the meaning of this regulation are aimed, in the context of the detection, monitoring, treatment, or alleviation of diseases or the detection, treatment, alleviation, or compensation of injuries or disabilities, at supporting patients' health-related actions or at integrating processes between patients and healthcare providers. They particularly include the following areas:<br>2. alignment of treatment with clinical guidelines and recognized standards,<br>3. adherence,<br>6. health literacy,<br>7. patient empowerment,<br>8. coping with disease-related challenges in everyday life,<br>[...]"
Overall Impression	Overall subjective rating, perceived utility, user trust	§ 139e Abs. 13 Nr. 2 SGB V "Zu den Ergebnissen der anwendungsbegleitenden Erfolgsmessung [...] zählen insbesondere [...] die Patientenzufriedenheit in Bezug auf die Qualität der digitalen Gesundheitsanwendung."	§ 139e (13) No. 2 SGB V: "The results of the accompanying evaluation of effectiveness [...] shall include, in particular, patient satisfaction with regard to the quality of the digital health application."

Results:

App and Statement Distribution

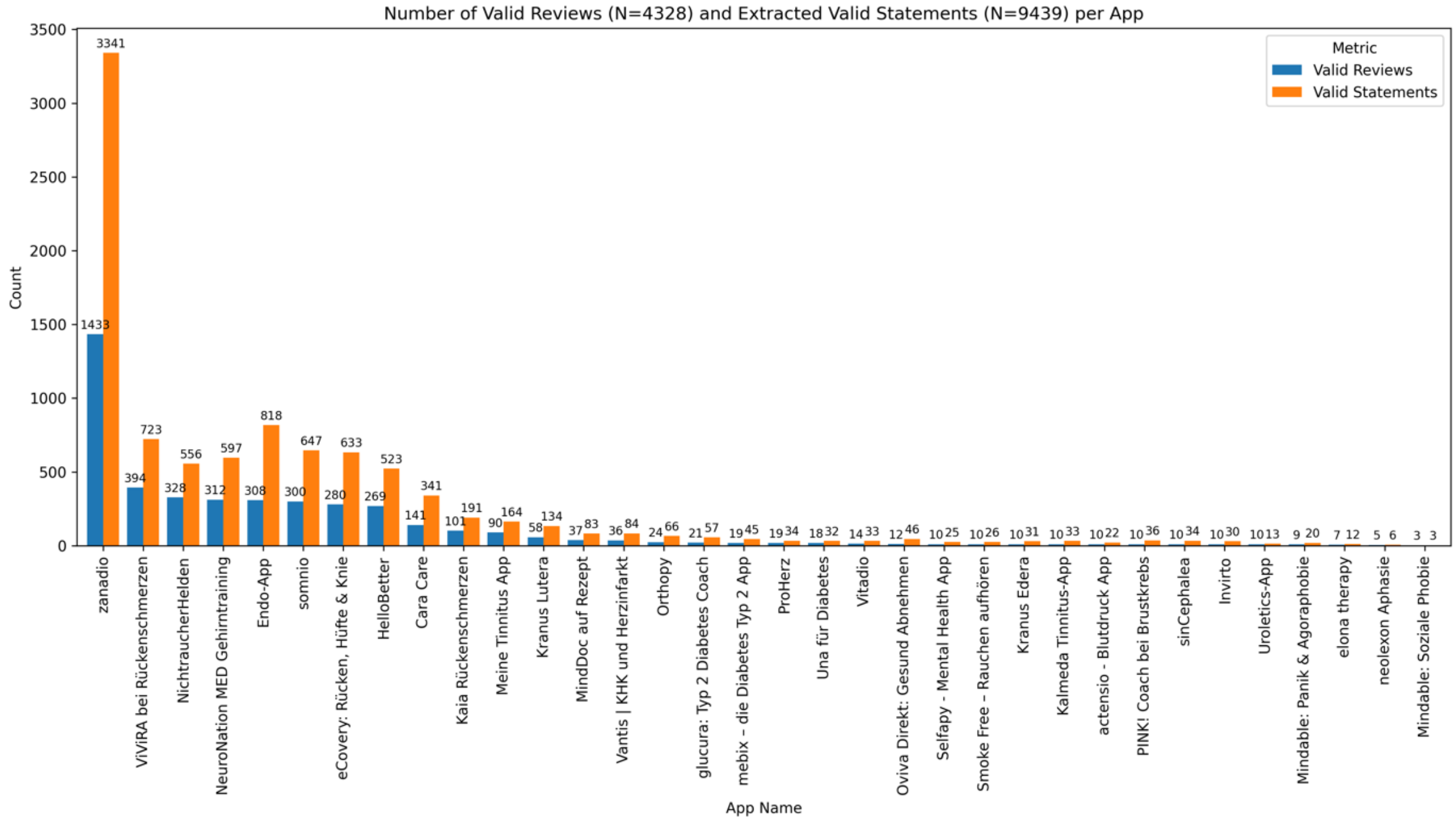

Figure 1: Number of Unique Reviews and Extracted Statements per App

Categories

Cost/Reimbursement

A total of 290 user statements addressed cost-related aspects or the reimbursement process via statutory health insurance. Users explicitly highlighted the positive financial accessibility of reimbursable DTx' through prescriptions. Some users valued that an application worked well and that the cost was covered by health insurance, while noting they might not pay for it themselves. Others expressed appreciation that an app helped them manage a chronic condition and improve disease understanding, with easy access to support and free provision via prescription.

Neutral statements acknowledged the benefit of reimbursement while expressing concerns about the overall price-performance relations, for instance that insurance covered most of the cost.

Critical statements often referred to high prices or complicated reimbursement procedures. Some users were satisfied with an application but considered the price too high and missed certain functions such as an offline mode. Others questioned pricing policies, pointing out that a reimbursed version cost their insurer substantially more than an apparently identical consumer subscription. Another user found that application helpful but described the recurring need to request a new access code every 30 days are burdensome, to the point of intending to

discontinue use. Similarly, another user questioned the cost-benefit ratio, noting that the exercise matched those from a low-cost book while the insurer paid a far higher price.

Content

The category Content received a total of 2,565 user statements. A large share of this feedback referred to the informational content as well as educational and therapeutic resources provided by the DTx's. Many users appreciated the diversity and practical value of the content, as well as the integration of scientifically based information. Some users described the content as clear and concice and valued the videos and the sense of support from daily feedback. Others characterized the underlying concepts as scientifically sound. Another user valued the varied, non-judgmental support delivered through topic-based modules covering areas such as exercise, nutrition, medication, relaxation, and smoking.

Neutral feedback highlighted positive aspects but also pointed to limitations, such as valuing the extensive knowledge and insurance coverage while preferring more audio content over text. In contrast, some reviews were explicitly critical regarding the app content. One user felt they had learned nothing new and doubted that limited information alone could change behavior. Another user expressed dissatisfaction with limited functionality and lack of new information, adding that data were deleted without a renewed prescription and that the symptom diary and exercises provided no personal benefit.

UX/Design

For the UX/Design category, 606 positive, 83 neutral and 340 negative statements in the dataset were allocated. Positive feedback highlighted the intuitive structure, visual presentation or accessibility of the evaluated DTx. Some users noted that condition-specific exercises were clearly explained through varied videos and that the app was intuitive to use. Others emphasized that an application was easy to understand and made working with their condition engaging. Further positive feedback described the modules as well explained and the application as very user-friendly.

Neutral statements often included suggestions for improvement without entirely negative sentiment, such as appreciating the guidance but wishing for a text-to-speech option.

Negative user statements focused on missing functionalities or technical limitation effecting their experience. One user criticized a small video display without full-screen or TV streaming options and reported that playback stopped after a few seconds. Other criticism addressed missing features such as a dark mode or timer functionality. Another user noted design-related

inconsistencies, reporting that some animations did not work although the application functioned reasonably well overall.

Tracking/ Documentation

For the Tracking/Documentation category, 230 positive, 43 neutral, and 195 negative statements in the dataset address the tracking and documentation functionalities of the analyzed applications. Positive feedback frequently highlighted the ability to gain structured insights into user's health status and progression. Some users valued that daily logging of food intake gave them a useful overview. Others appreciated that all content, including mood tracking, was now available within the mobile app rather than only the web version.

Neutral reviews often suggested improvements, especially regarding the scope of tracking options, for example finding the app motivating but withing for additional trackable values and more practical recipe ideas.

Negative feedback mainly criticized technical shortcomings or usability barriers related to tracking functionalities. One user reported frequent interrupting notifications during tracking and an inability to enter meals with more than five ingredients. Another user criticized unreliable data integration, including failed synchronization with an external fitness tracker.

Technology

A total of 668 user statements in the dataset address the technical performance and stability of the analyzed applications. Positive feedback emphasized smooth functionality and reliable operation. Some users reported that an application ran flawlessly, with clear explanations and easy-to-follow exercises. Others confirm that an application ran very stably for them and found that courses helpful.

Neutral feedback acknowledged minor technical issues while still rating the application as helpful, regarding occasional glitches as negligible.

Negative user feedback mainly criticized crashes, slow performance, or severe malfunctions. One user reported that, following successive updates, the application became sluggish and then repeatedly logged them out, with entries disappearing, rendering it barely usable. Another user described a more fundamental problem in which the application closed immediately after the start screen and could not be opened even after reinstallation.

Support

In total, 865 user statements were assigned to the category Support, addressing user experiences with both technical assistance and supportive features such as guided sessions or communication with healthcare professionals. Many users expressed satisfaction with the

availability and quality of support services. Some users described the customer support as excellent. Others valued both the convenience of documenting everything in one app and the quality of the support team.

However, technical issues and delayed responses were also reported. One user described ongoing login problems and crashes that support had been working on for weeks, while still describing the chat support as friendly and responsive.

Negative experiences often related to unresolved technical problems or insufficient assistance. One user reported that several features did not work and that support could not help, which they considered unacceptable given the price. Another user criticized a lack of response, reporting a three-month wait for an answer to a question.

Prescription/ Approval

A total of 234 user statements were assigned to the category Prescription/ Approval, reflecting user experiences with obtaining or redeeming access codes for the DTx. Positive Feedback emphasized the convenience and accessibility of the prescription process. Some users described quick and reliable access to prescriptions as making everyday life easier. Others valued the combination of medical support and accessibility, noting the unusual opportunity to obtain a personal coach by prescription.

Neutral feedback highlighted mixed experiences, particularly regarding bureaucratic hurdles, such as finding an application helpful but describing the multistep process of obtaining and renewing the access code as complicated.

Other users criticized the lack of information about the prescription, noting that the application was unusable without a prescription code and that their physician was unaware of it, objecting that they could not test the application before completing the required paperwork.
