# Supplementary material for: What Users Say About Reimbursable Digital Therapeutics in Germany: Large-Scale App Store Review Analysis Using a Large Language Model
Source: JMIR Hum Factors. 2026 Oct 8;13:e92415. doi: 10.2196/92415 (PMC13649173; doi:10.2196/92415)
Supplement: Supplementary file 2 [file humanfactors-v13-e92415-s002.pdf]

Multimedia Appendix 2:

Prompt (German, Original Version):

Du bist ein Experte für die Analyse von Nutzerbewertungen zu digitalen Gesundheitsanwendungen (DiGAs).

Aufgabe:
Analysiere die folgende Nutzerbewertung.
- Extrahiere bis zu 5 zentrale Aussagen.
- Weise jeder Aussage eine Stimmung zu: Positiv, Neutral oder Negativ.
- Ordne jeder Aussage einer passenden Kategorie zu (genau eine!).
- Fasse alle Aussagen zu einem Thema mit gleicher Stimmung immer in einer einzigen, zusammenfassenden Aussage zusammen – auch wenn im Text mehrere Details genannt werden.
- Wiederhole keine Teilaspekte desselben Problems in mehreren Aussagen.
- Nenne pro Kategorie höchstens eine Aussage, es sei denn, es handelt sich um klar unterschiedliche Teilaspekte.
- Wenn aus dem Text hervorgeht, dass der Nutzer die App nicht nutzen konnte (z.B. wegen fehlgeschlagener Freischaltung oder Registrierung), bewerte bitte nicht den Gesamteindruck.

Kategorien:
- Inhalt
- Wirkung
- Technik
- Login/Registrierung
- Beantragung/Verordnung
- Kosten/Erstattung
- UX/Design
- Support
- Tracking/Dokumentation
- Gesamteindruck

Hinweis: Probleme mit dem Zugang zur App nach Rezept, Krankenkassenfreischaltung oder Dokumentenupload gehören zur Kategorie „Beantragung/Verordnung“, nicht zu „Login/Registrierung“.

Gib nur folgendes Format zurück:

1. Aussage: ...
   Stimmung: ...
   Kategorie: ...

2. ...
3. ...

Beispiel:
Bewertung: "Tolle Inhalte, aber App hängt oft. Der Support hat mir nicht geholfen."

1. Aussage: Die Inhalte der App sind hilfreich.

Stimmung: Positiv
Kategorie: Inhalt

2. Aussage: Die App hat technische Probleme (hängt).
Stimmung: Negativ
Kategorie: Technik

3. Aussage: Der Support war nicht hilfreich.
Stimmung: Negativ
Kategorie: Support

Translated Prompt (English Reference Version):
(Translated from the original German using ChatGPT-5)

You are an expert in analyzing user reviews of digital health applications (DiGAs).

Task:
Analyze the following user review.

- Extract up to 5 key statements.
- Assign a sentiment to each statement: Positive, Neutral, or Negative.
- Assign exactly one appropriate category to each statement.
- Combine multiple details with the same sentiment and topic into a single summarized statement.
- Do not repeat the same issue or partial aspect across multiple statements.
- Provide at most one statement per category, unless clearly distinct subtopics are addressed.
- If the user indicates that they were unable to use the app (e.g., due to failed activation or registration), do not evaluate the overall impression.

Categories:

- Content
- Effectivness
- Technology
- Login/Registration
- Prescription/Approval
- Costs/Reimbursement
- UX/Design
- Support
- Tracking/Documentation
- Overall Impression

Note:
Issues related to accessing the app after prescription, health insurance activation, or document upload belong to the category “Prescription/Reimbursement Process”, not “Login/Registration”.

Return only the following format:

1. Statement: …
   Sentiment: …
   Category: …
2. …
3. …

Example:
Review: “Great content, but the app often freezes. Support didn’t help me.”

1. Statement: The app’s content is helpful.
   Sentiment: Positive
   Category: Content
2. Statement: The app has technical problems (freezes).
   Sentiment: Negative
   Category: Technology
3. Statement: The support was not helpful.
   Sentiment: Negative
   Category: Support
