# Supplementary material for: What Users Say About Reimbursable Digital Therapeutics in Germany: Large-Scale App Store Review Analysis Using a Large Language Model
Source: JMIR Hum Factors. 2026 Oct 8;13:e92415. doi: 10.2196/92415 (PMC13649173; doi:10.2196/92415)
Supplement: Supplementary file 3 [file humanfactors-v13-e92415-s003.pdf]

Multimedia Appendix 3: Topic Modelling Output (BERTopic)

As an exploratory alternative to the predefined statement-level classification, unsupervised topic modelling was applied to 4,270 unique user reviews using BERTopic with a multilingual sentence transformer model (paraphrase-multilingual-mpnet-base-v2).

The analysis yielded 67 topics, while 1,372 reviews (32.1%) could not be assigned to any topic. The topics primarily reflected indications and individual applications rather than the quality dimensions relevant for regulatory assessment. Each topic covered a median of 8 of the ten predefined categories, and the most frequent category accounted for a median of 39% of a topic's statements, indicating that the topics could not be mapped consistently onto the regulatory framework.

Topics

Topics with at least 40 documents are listed individually. Topic -1 contains reviews that could not be assigned to any topic. "Categories" indicates how many of the ten predefined categories are represented among a topic's statements; "Dominant (%)" is the share of statements falling into the most frequent category. 45 smaller topics (967 documents in total) are not listed individually.

Topic ID	Documents	Top terms	Categories	Dominant (%)	Dominant category
-1	1372	die, und, ich, nicht, app, der	10	24.3	Content
0	166	übungen, gut, gute, erklärt, sind, sehr	9	54.2	Content
1	135	erklärt, sehr, verständlich, gut, informativ, bedienen	7	49.7	Content
2	129	symptome, app, die, und, ich, sehr	10	27.1	Content
3	119	krankenkasse, nicht, geld, die, für, preis	10	30.7	Cost/Reimbursement
4	118	app, tolle, gute, sehr, super, empfehlen	7	59.7	Overall Impression
5	116	rauchen, rauchfrei, zigaretten, geraucht, rauchstopp, habe	9	38.9	Effectiveness

6	114	zanadio, und, bin, ich, das, team	9	29.3	Content
7	114	schlaf, hat, das, ich, app, zu	10	25.4	Effectiveness
8	112	chat, sehr, super, fragen, app, tolle	9	30.6	Support
9	110	ernährung, und, bewegung, bin, ich, habe	9	34.3	Content
10	103	kg, ernährung, und, app, mit, abnehmen	10	27.0	Effectiveness
11	69	gedächtnis, kognitiven, neuronation, kognitive, covid, training	8	41.2	Effectiveness
12	69	hilfreich, geholfen, hilft, sehr, funktioniert, hat	8	40.0	Effectiveness
13	65	nette, kompetente, beratung, gute, ansprechpartner, freundliche	5	49.1	Support
14	64	übungen, übung, die, nicht, nach, werden	10	25.9	Content
15	56	übungen, erklärt, gut, werden, app, sehr	8	44.0	Content
16	53	super, alles, gut, okay, einfach, mega	4	87.0	Overall Impression
17	50	endometriose, die, und, für, der, app	10	33.1	Content
18	44	bin, dabei, erst, jetzt, eindruck, anfang	6	65.4	Overall Impression
19	43	zufrieden, bin, sehr, begeistert, frieden, fühle	5	68.0	Overall Impression
20	42	lebensmittel, man, mahlzeit, eintragen, könnte, der	9	24.1	UX/Design
21	40	tinnitus, den, damit, stimme, besser, ich	8	37.7	Content

Note: German stop words were not removed prior to topic modelling, which is reflected in the top terms of several topics.
