# Supplementary material for: What Users Say About Reimbursable Digital Therapeutics in Germany: Large-Scale App Store Review Analysis Using a Large Language Model
Source: JMIR Hum Factors. 2026 Oct 8;13:e92415. doi: 10.2196/92415 (PMC13649173; doi:10.2196/92415)
Supplement: Supplementary file 4 [file humanfactors-v13-e92415-s005.pdf]

Multimedia Appendix 5: Clustering-Adjusted Analysis

Because user statements originating from the same application are unlikely to be fully independent, all analyses were repeated using an app-level averaging approach. In this clustering-adjusted analysis, sentiment proportions were first calculated separately for each application and then averaged across applications, so that every application contributed equally regardless of the number of statements it provided. This appendix reports both the pooled (statement-weighted) and the clustering-adjusted (app-averaged) results for comparison.

Full Dataset (All Applications)

Statements: 9,439 | Applications: 34

Overall Sentiment Distribution

Sentiment	Pooled (%)	App-averaged (%)	Difference (pp)
Positiv	68.8	64.5	-4.3
Neutral	5.0	5.4	0.4
Negativ	26.2	30.0	3.8

Positive Statements by Category

Category	Pooled Positiv (%)	App-averaged Positiv (%)	Difference (pp)
Content	78.0	72.6	-5.4
Cost/Reimbursement	27.2	40.7	13.5
Effectiveness	89.5	90.5	1.0
Login/Registration	5.0	12.0	7.0
Overall Impression	86.2	80.4	-5.8
Prescription/Approval	25.2	31.3	6.1
Support	87.4	73.2	-14.2
Technology	10.9	15.7	4.8
Tracking/Documentation	49.1	49.6	0.5
UX/Design	58.9	61.5	2.6

Negative Statements by Category

Category	Pooled Negativ (%)	App-averaged Negativ (%)	Difference (pp)
Content	16.3	20.1	3.8
Cost/Reimbursement	66.9	54.4	-12.5
Effectiveness	6.3	6.5	0.2
Login/Registration	93.9	86.7	-7.2
Overall Impression	10.3	13.3	3.0
Prescription/Approval	65.4	59.7	-5.7
Support	11.3	23.6	12.3
Technology	84.3	80.5	-3.8
Tracking/Documentation	41.7	40.7	-1.0
UX/Design	33.0	34.0	1.0

Category Size and Application Coverage

The number of statements per category and the median number of statements per application indicate how stable the app-averaged estimates are. Categories with few statements per application are more sensitive to individual reviews.

Category	Statements	Apps	Median statements per app
Content	2565	32	14.0
Cost/Reimbursement	290	30	3.5
Effectiveness	1609	33	11.0
Login/Registration	280	19	6.0
Overall Impression	1431	34	5.5
Prescription/Approval	234	22	3.5
Support	865	25	5.0
Technology	668	29	6.0
Tracking/Documentation	468	27	4.0
UX/Design	1029	32	8.0

Graphical Comparison

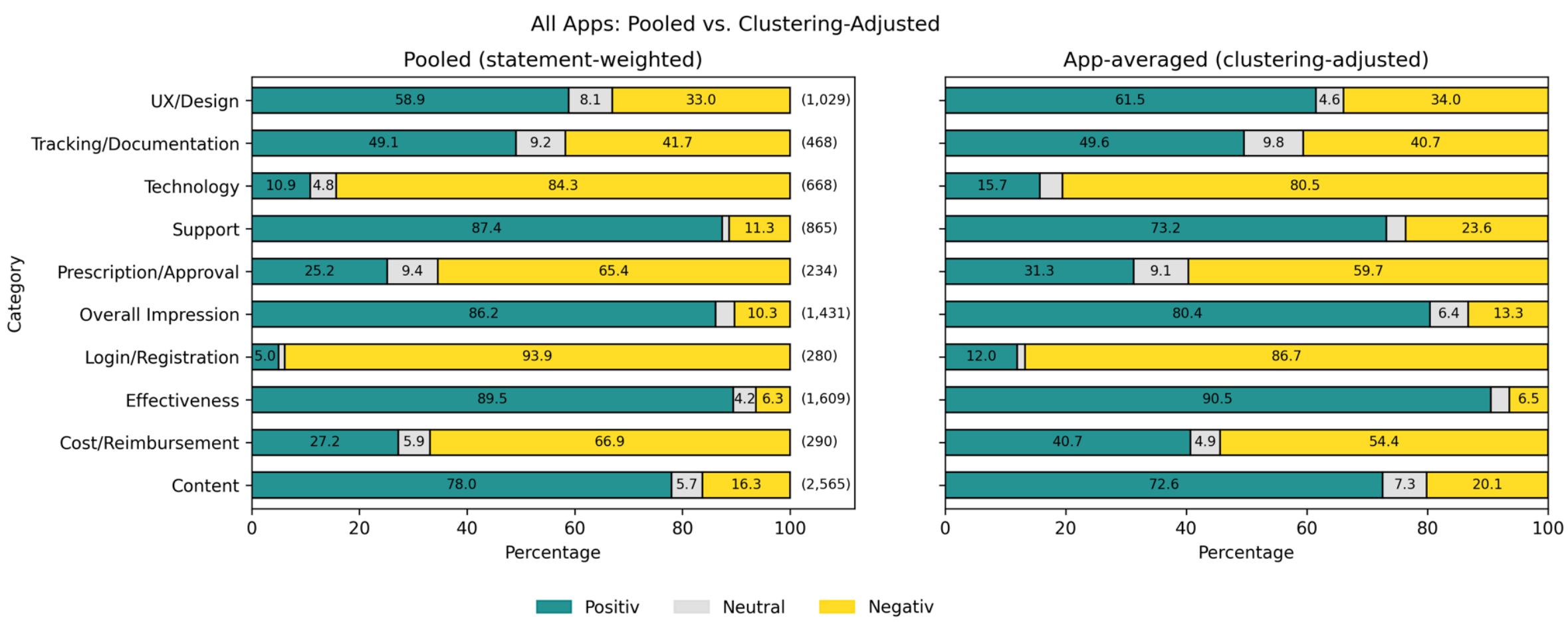

Sentiment distribution per category, pooled versus clustering-adjusted (Full Dataset (All Applications)).

Sensitivity Analysis (Excluding Zanadio)

Statements: 6,098 | Applications: 33

Overall Sentiment Distribution

Sentiment	Pooled (%)	App-averaged (%)	Difference (pp)
Positiv	65.0	64.2	-0.8
Neutral	5.6	5.5	-0.1
Negativ	29.5	30.3	0.8

Positive Statements by Category

Category	Pooled Positiv (%)	App-averaged Positiv (%)	Difference (pp)
Content	73.4	72.1	-1.3

Cost/Reimbursement	26.1	41.1	15.0
Effectiveness	88.6	90.4	1.8
Login/Registration	6.4	12.6	6.2
Overall Impression	85.7	80.2	-5.5
Prescription/Approval	28.0	31.9	3.9
Support	66.0	72.3	6.3
Technology	9.2	15.7	6.5
Tracking/Documentation	58.2	50.3	-7.9
UX/Design	56.4	61.4	5.0

Negative Statements by Category

Category	Pooled Negativ (%)	App-averaged Negativ (%)	Difference (pp)
Content	20.1	20.5	0.4
Cost/Reimbursement	66.1	53.8	-12.3
Effectiveness	7.3	6.6	-0.7
Login/Registration	92.6	86.1	-6.5
Overall Impression	10.8	13.4	2.6
Prescription/Approval	60.1	58.8	-1.3
Support	30.2	24.4	-5.8
Technology	87.1	80.7	-6.4
Tracking/Documentation	33.9	40.0	6.1
UX/Design	35.4	34.2	-1.2

Category Size and Application Coverage

The number of statements per category and the median number of statements per application indicate how stable the app-averaged estimates are. Categories with few statements per application are more sensitive to individual reviews.

Category	Statements	Apps	Median statements per app
Content	1674.0	31.0	13.0
Cost/Reimbursement	218.0	29.0	3.0
Effectiveness	1180.0	32.0	10.5
Login/Registration	204.0	18.0	5.5
Overall Impression	942.0	33.0	5.0
Prescription/Approval	168.0	21.0	3.0
Support	212.0	24.0	4.0
Technology	480.0	28.0	5.0
Tracking/Documentation	316.0	26.0	4.0
UX/Design	704.0	31.0	8.0

Graphical Comparison

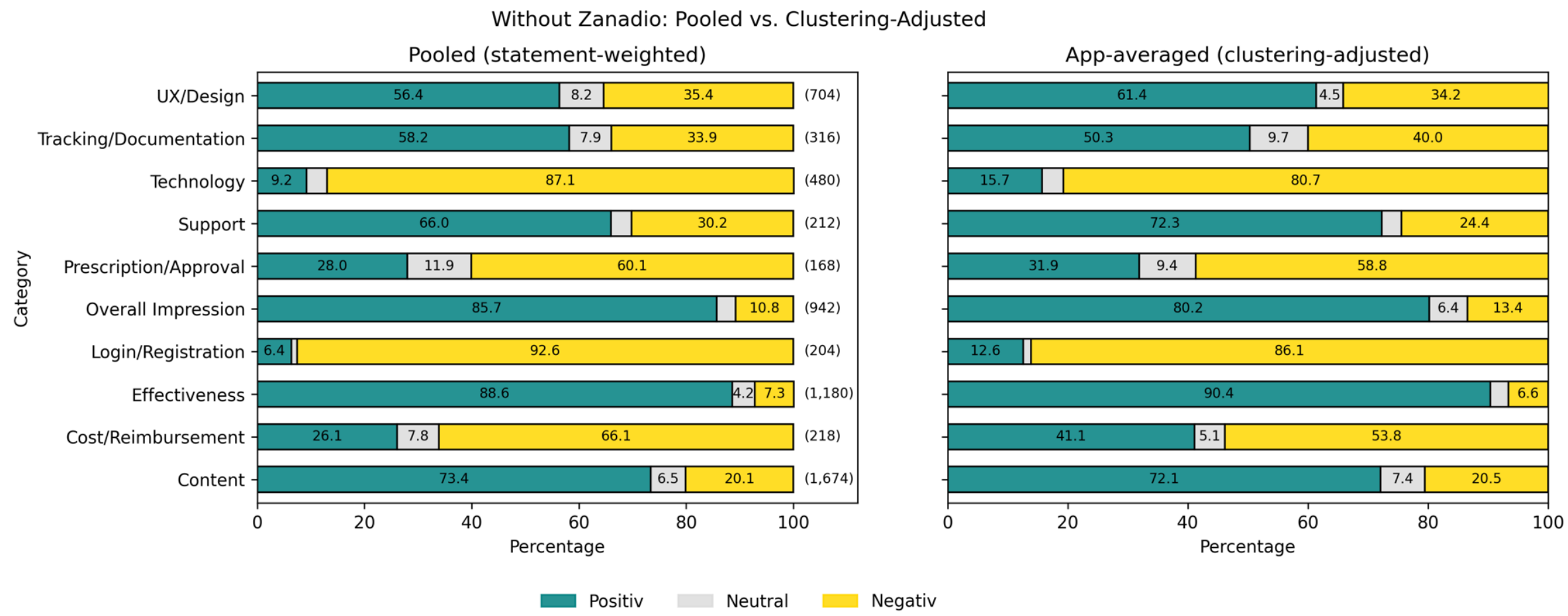

Sentiment distribution per category, pooled versus clustering-adjusted (Sensitivity Analysis (Excluding Zanadio)).
