# Supplementary material for: What Users Say About Reimbursable Digital Therapeutics in Germany: Large-Scale App Store Review Analysis Using a Large Language Model
Source: JMIR Hum Factors. 2026 Oct 8;13:e92415. doi: 10.2196/92415 (PMC13649173; doi:10.2196/92415)
Supplement: Supplementary file 5 [file humanfactors-v13-e92415-s006.pdf]

Appendix III:
Category and Sentiment Analysis per App

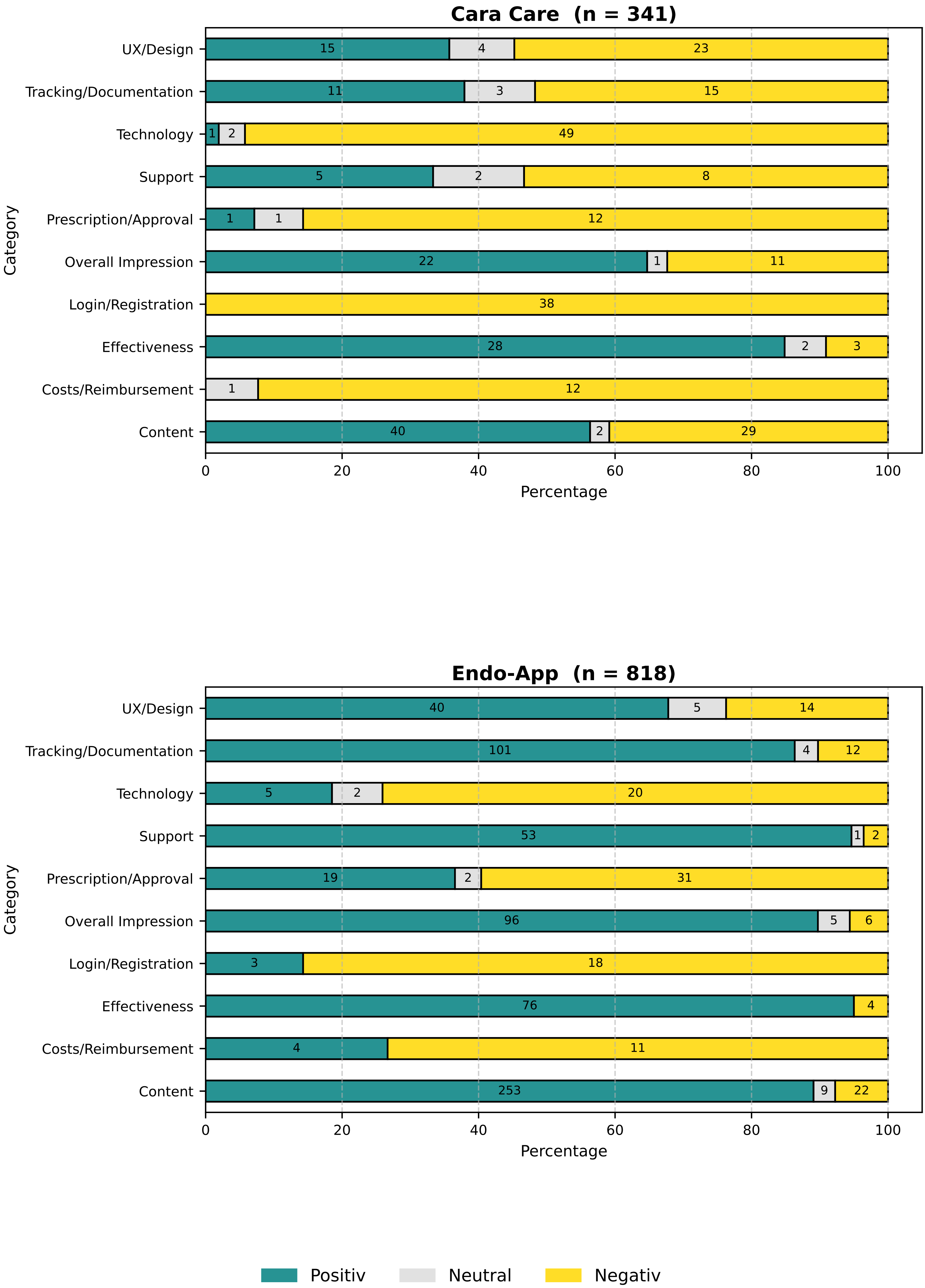
Cara Care (n = 341)
UX/Design
Tracking/Documentation
Technology
Support
Prescription/Approval
Overall Impression
Login/Registration
Effectiveness
Costs/Reimbursement
Content
Category
Percentage
15 4 23
11 3 15
1 2 49
5 2 8
1 1 12
22 1 11
38
28 2 3
1 12
40 2 29
0 20 40 60 80 100
Endo-App (n = 818)
UX/Design
Tracking/Documentation
Technology
Support
Prescription/Approval
Overall Impression
Login/Registration
Effectiveness
Costs/Reimbursement
Content
Category
Percentage
40 5 14
101 4 12
5 2 20
53 1 2
19 2 31
96 5 6
3 18
76 4
4 11
253 9 22
0 20 40 60 80 100
Positiv
Neutral
Negativ

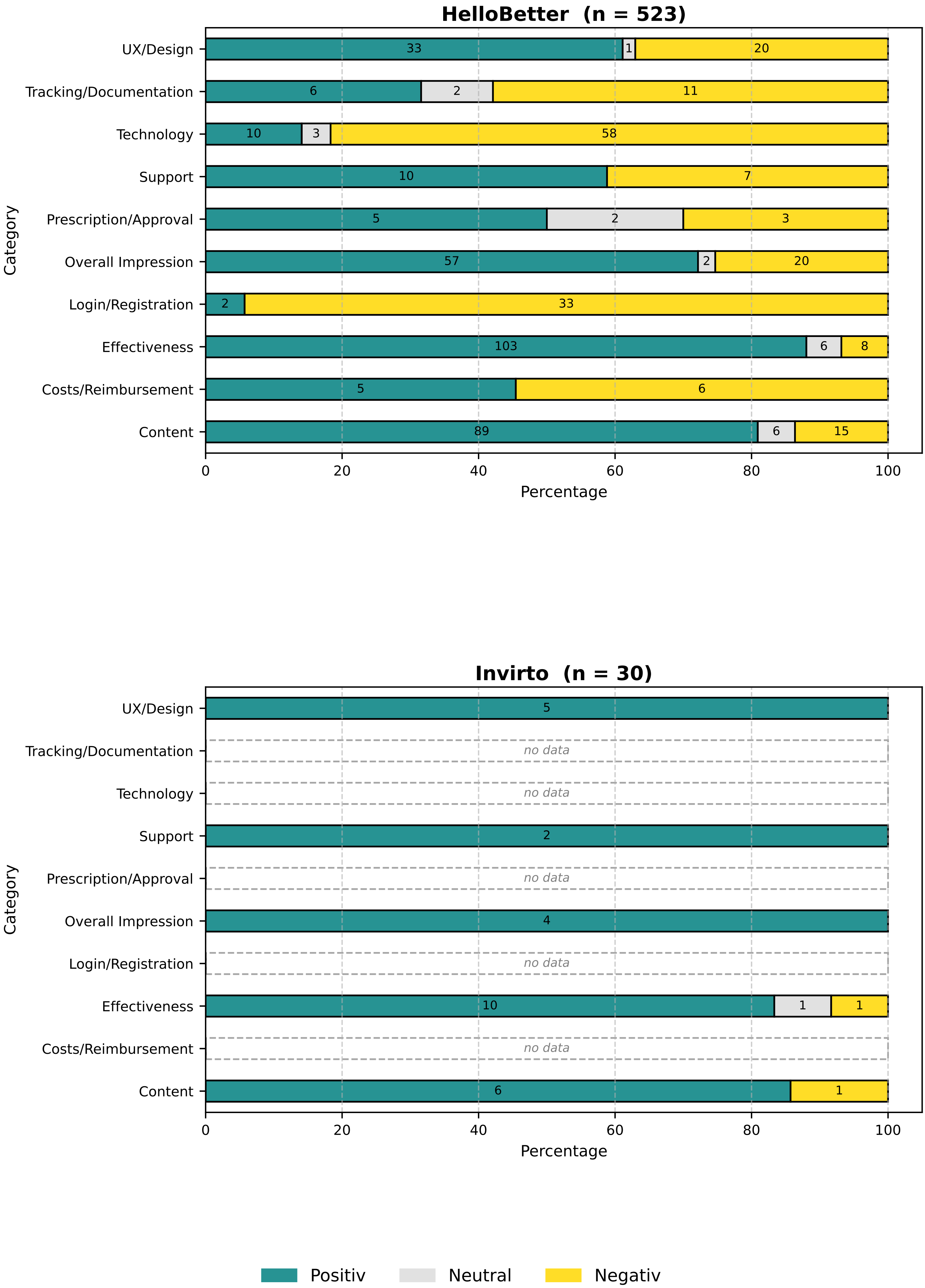
HelloBetter (n = 523)
UX/Design
Tracking/Documentation
Technology
Support
Prescription/Approval
Overall Impression
Login/Registration
Effectiveness
Costs/Reimbursement
Content
Category
Percentage
33 1 20
6 2 11
10 3 58
10 7
5 2 3
57 2 20
2 33
103 6 8
5 6
89 6 15
0 20 40 60 80 100
Invirto (n = 30)
UX/Design
Tracking/Documentation
Technology
Support
Prescription/Approval
Overall Impression
Login/Registration
Effectiveness
Costs/Reimbursement
Content
Category
Percentage
5
no data
no data
2
no data
4
no data
10 1 1
no data
6 1
0 20 40 60 80 100
Positiv
Neutral
Negativ

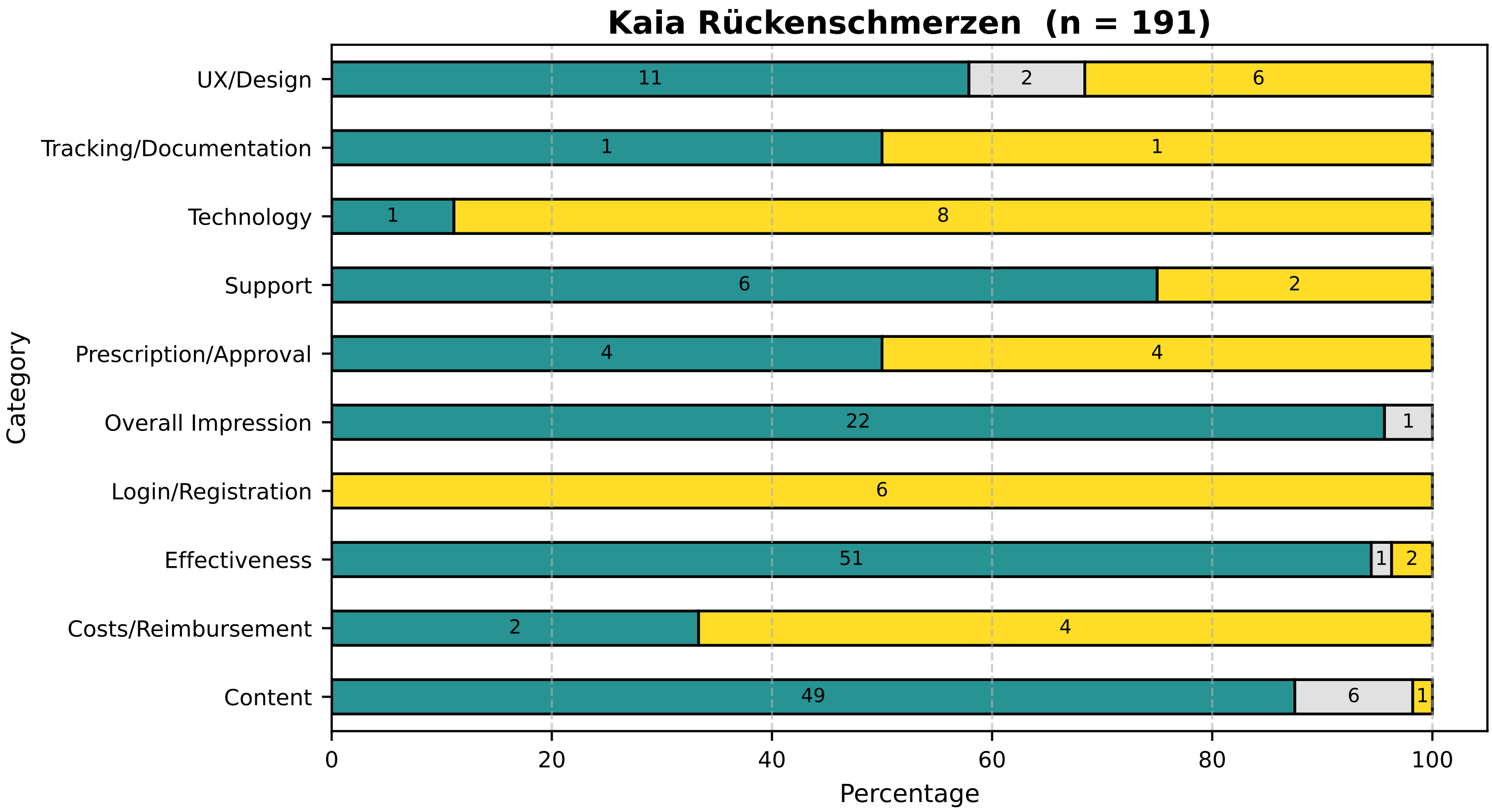

Kaia Rückenschmerzen (n = 191)
Category
UX/Design
Tracking/Documentation
Technology
Support
Prescription/Approval
Overall Impression
Login/Registration
Effectiveness
Costs/Reimbursement
Content
11 2 6
1 1
1 8
6 2
4 4
22 1
6
51 1 2
2 4
49 6 1
0 20 40 60 80 100
Percentage

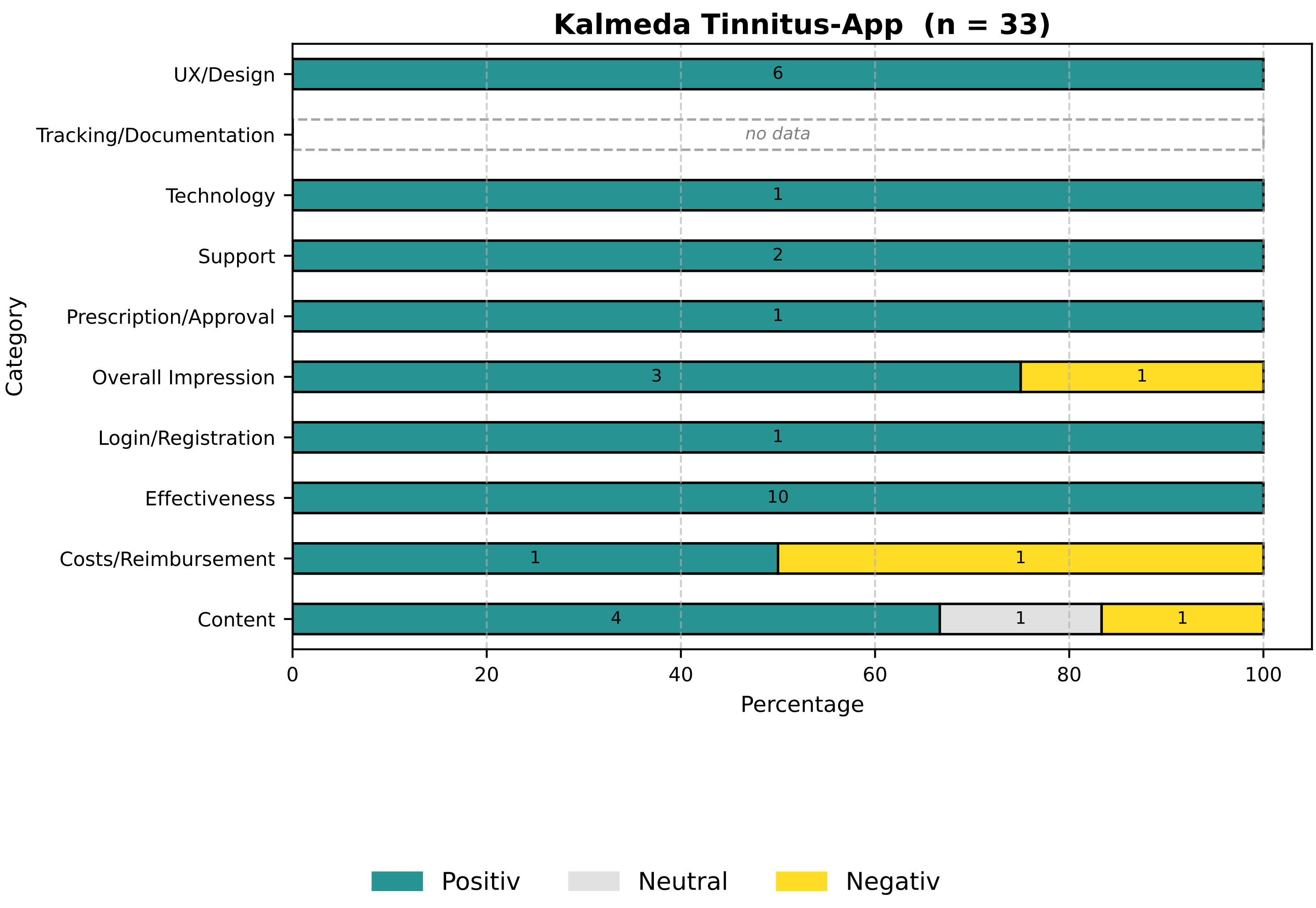

Kalmeda Tinnitus-App (n = 33)
Category
UX/Design
Tracking/Documentation
Technology
Support
Prescription/Approval
Overall Impression
Login/Registration
Effectiveness
Costs/Reimbursement
Content
6
no data
1
2
1
3 1
1
10
1 1
4 1 1
0 20 40 60 80 100
Percentage
Positiv
Neutral
Negativ

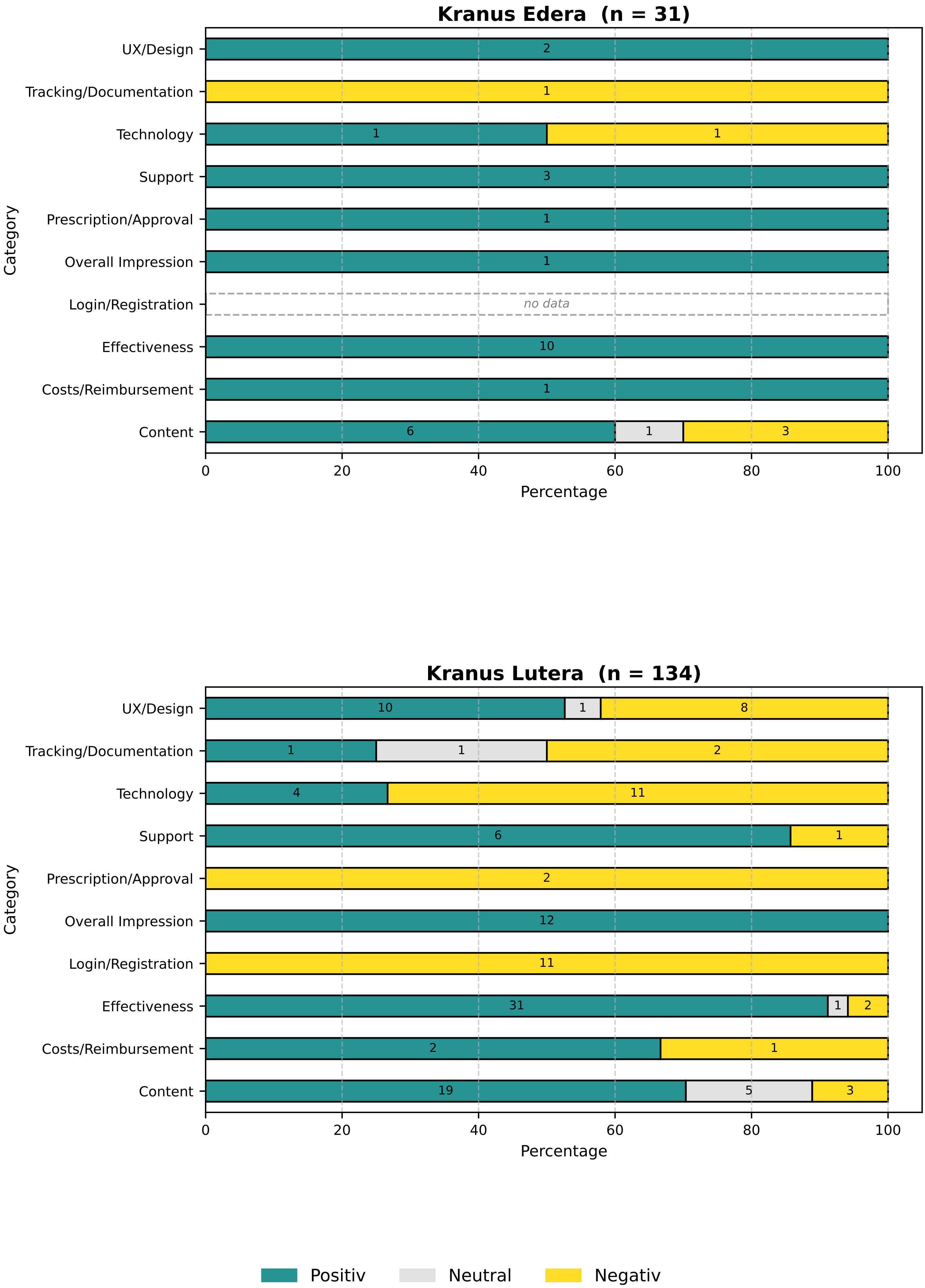
Kranus Edera (n = 31)
Category
UX/Design
Tracking/Documentation
Technology
Support
Prescription/Approval
Overall Impression
Login/Registration
Effectiveness
Costs/Reimbursement
Content
2
1
1
1
3
1
1
no data
10
1
6
1
3
0
20
40
60
80
100
Percentage
Kranus Lutera (n = 134)
Category
UX/Design
Tracking/Documentation
Technology
Support
Prescription/Approval
Overall Impression
Login/Registration
Effectiveness
Costs/Reimbursement
Content
10
1
8
1
1
2
4
11
6
1
2
12
11
31
1
2
2
1
19
5
3
0
20
40
60
80
100
Percentage
Positiv
Neutral
Negativ

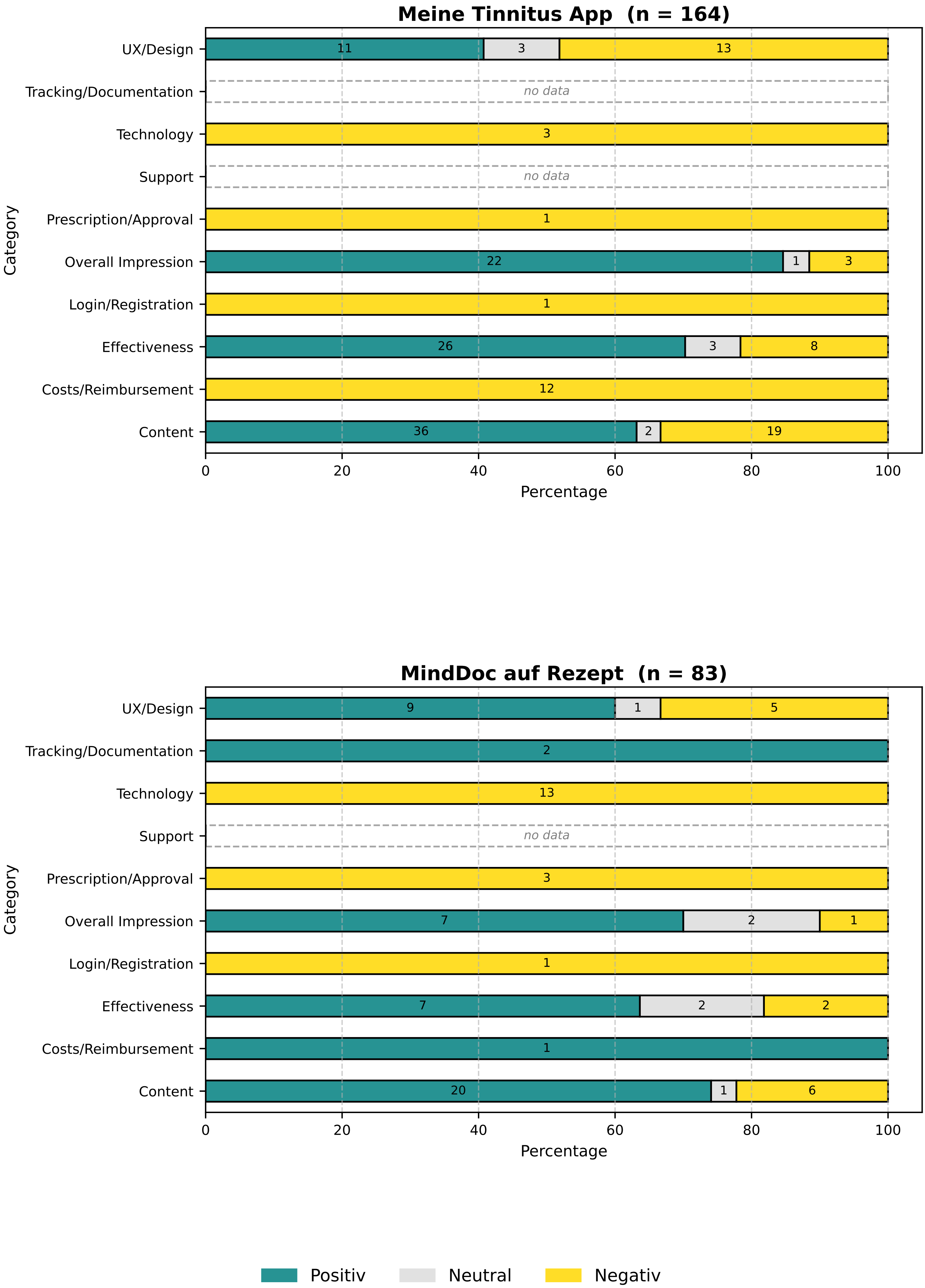

Meine Tinnitus App (n = 164)
UX/Design
Tracking/Documentation
Technology
Support
Prescription/Approval
Overall Impression
Login/Registration
Effectiveness
Costs/Reimbursement
Content
Category
Percentage
11
3
13
no data
3
no data
1
22
1
3
1
26
3
8
12
36
2
19
0
20
40
60
80
100
MindDoc auf Rezept (n = 83)
UX/Design
Tracking/Documentation
Technology
Support
Prescription/Approval
Overall Impression
Login/Registration
Effectiveness
Costs/Reimbursement
Content
Category
Percentage
9
1
5
2
13
no data
3
7
2
1
1
7
2
2
1
20
1
6
0
20
40
60
80
100
Positiv
Neutral
Negativ

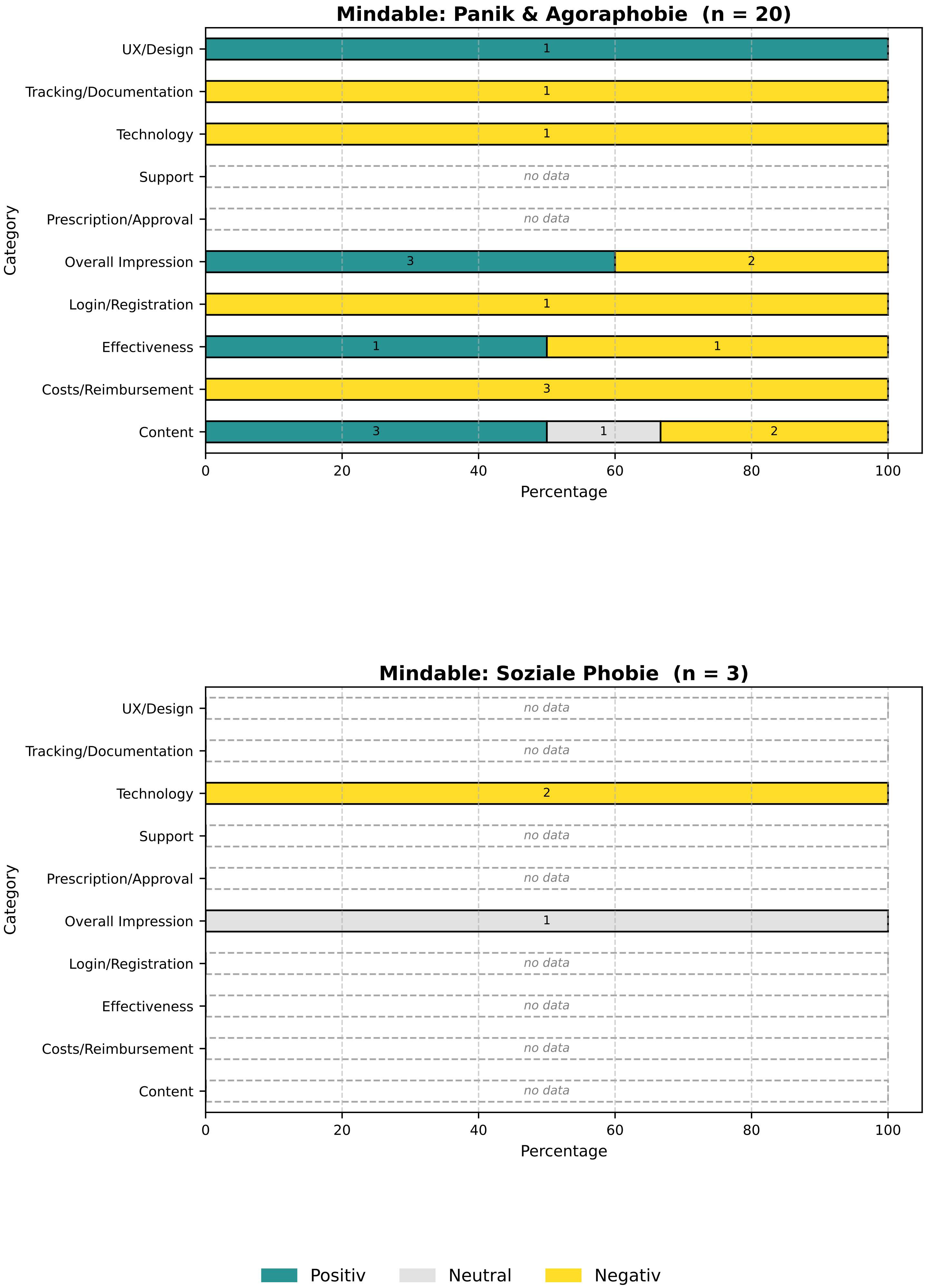
Mindable: Panik & Agoraphobie (n = 20)
UX/Design
Tracking/Documentation
Technology
Support
Prescription/Approval
Overall Impression
Login/Registration
Effectiveness
Costs/Reimbursement
Content
Category
Percentage
0
20
40
60
80
100
1
1
1
no data
no data
3
2
1
1
1
3
3
1
2
Mindable: Soziale Phobie (n = 3)
UX/Design
Tracking/Documentation
Technology
Support
Prescription/Approval
Overall Impression
Login/Registration
Effectiveness
Costs/Reimbursement
Content
Category
Percentage
0
20
40
60
80
100
no data
no data
2
no data
no data
1
no data
no data
no data
no data
Positiv
Neutral
Negativ

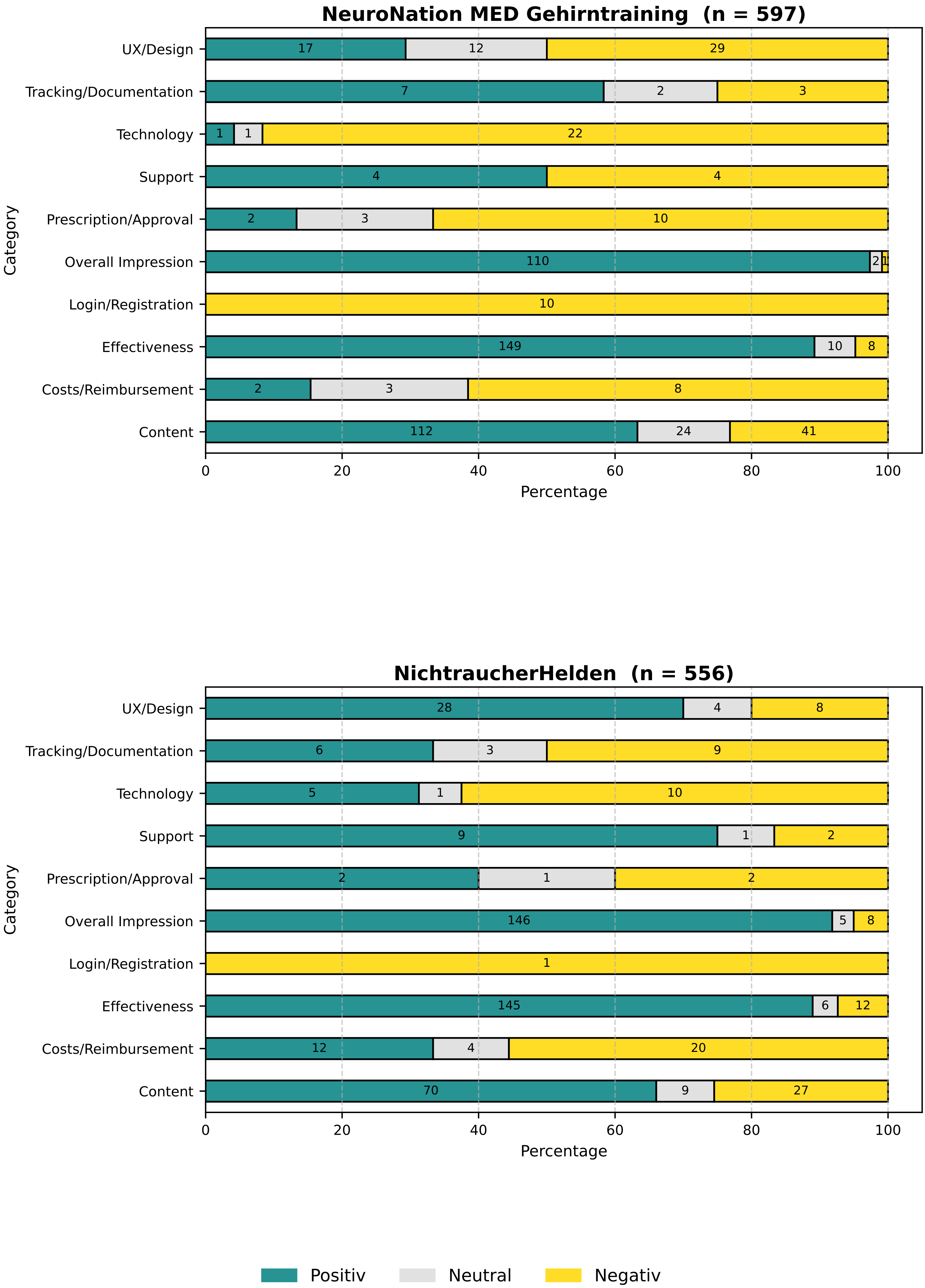
NeuroNation MED Gehirntraining (n = 597)
UX/Design 17 12 29
Tracking/Documentation 7 2 3
Technology 1 1 22
Support 4 4
Prescription/Approval 2 3 10
Overall Impression 110 2 1
Login/Registration 10
Effectiveness 149 10 8
Costs/Reimbursement 2 3 8
Content 112 24 41
Category
0 20 40 60 80 100
Percentage
NichtraucherHelden (n = 556)
UX/Design 28 4 8
Tracking/Documentation 6 3 9
Technology 5 1 10
Support 9 1 2
Prescription/Approval 2 1 2
Overall Impression 146 5 8
Login/Registration 1
Effectiveness 145 6 12
Costs/Reimbursement 12 4 20
Content 70 9 27
Category
0 20 40 60 80 100
Percentage
Positiv
Neutral
Negativ

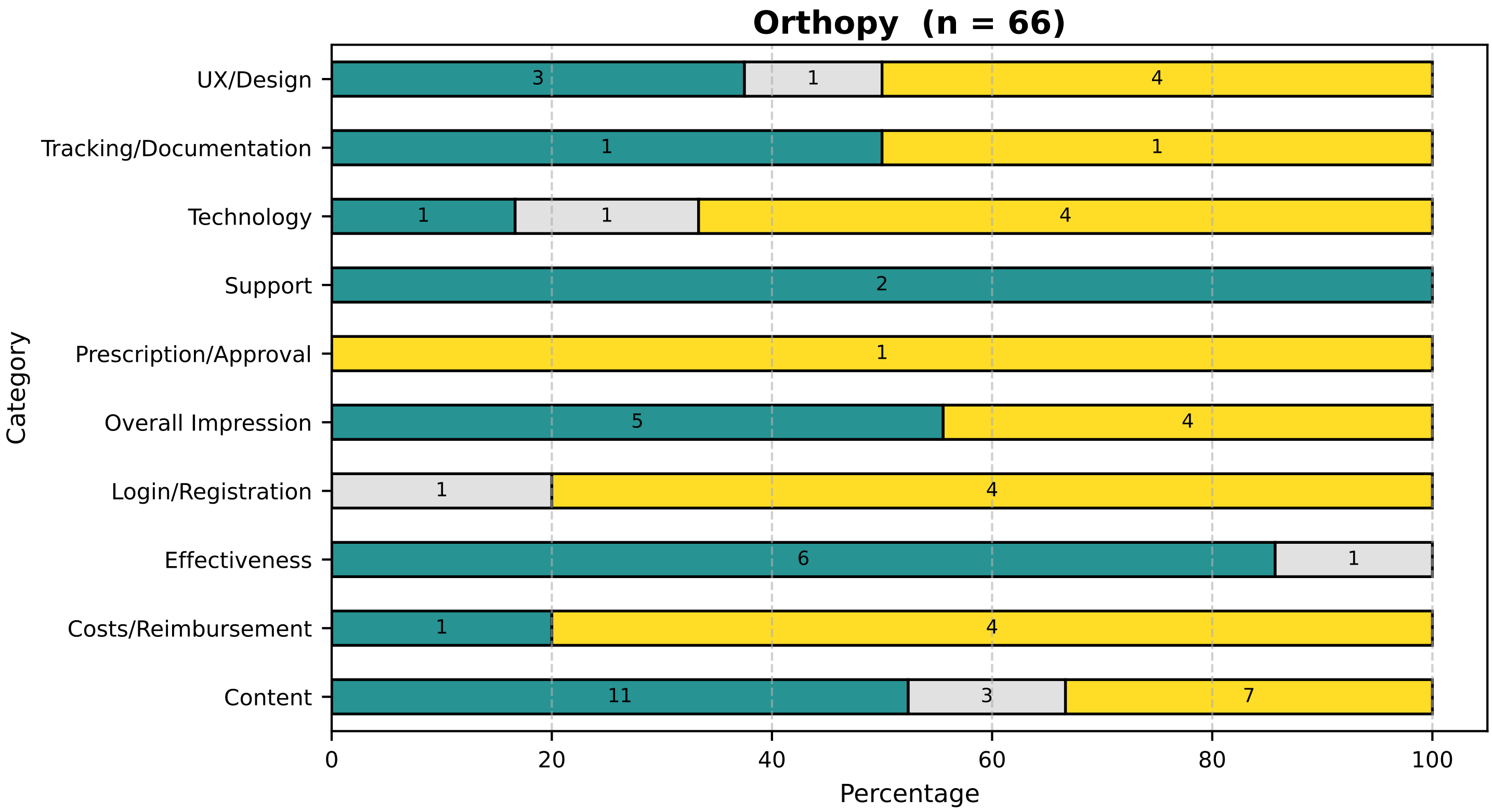

Orthopy (n = 66)
Category
UX/Design
Tracking/Documentation
Technology
Support
Prescription/Approval
Overall Impression
Login/Registration
Effectiveness
Costs/Reimbursement
Content
3
1
4
1
1
1
1
4
2
1
5
4
1
4
6
1
1
4
11
3
7
0
20
40
60
80
100
Percentage

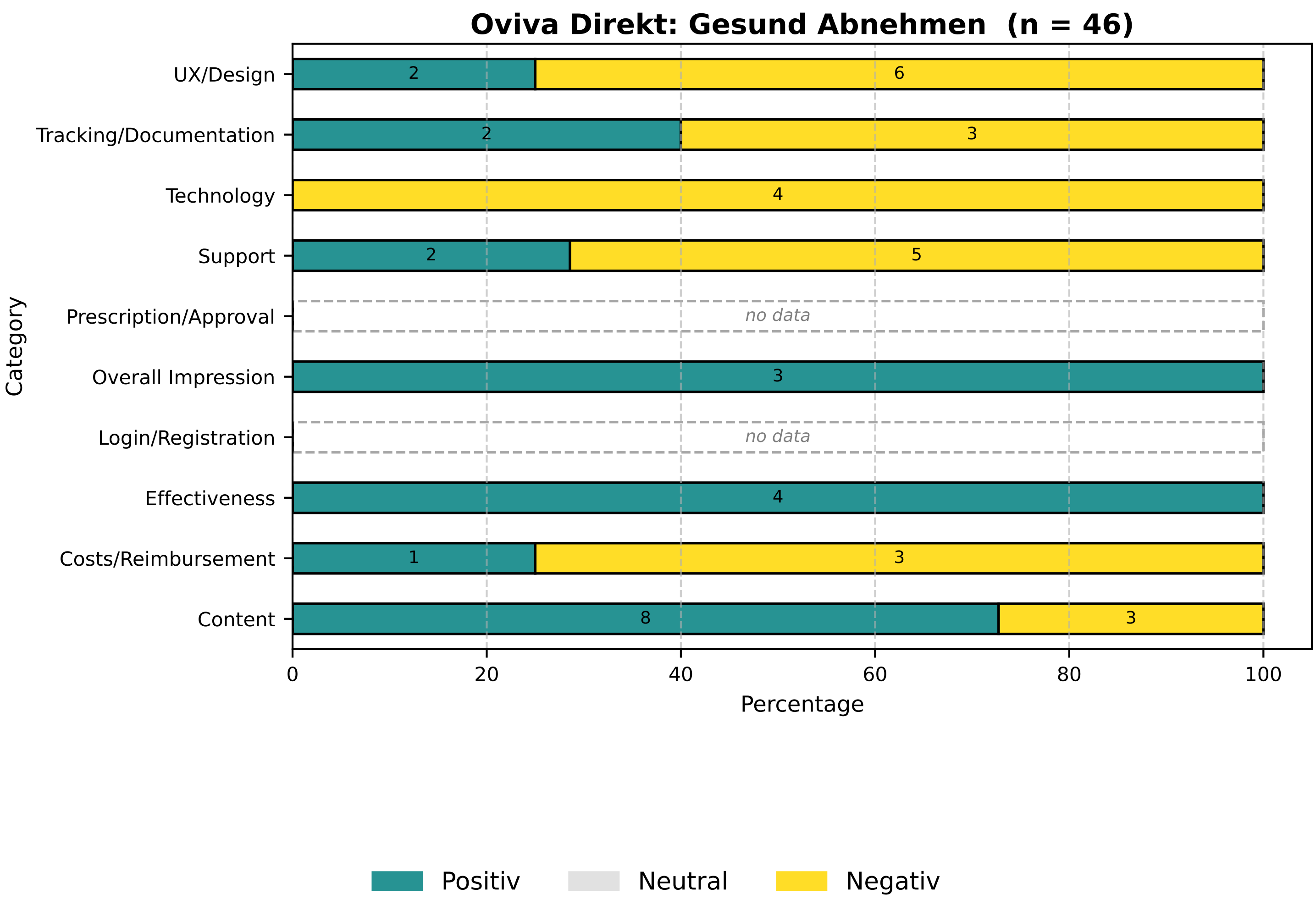

Oviva Direkt: Gesund Abnehmen (n = 46)
Category
UX/Design
Tracking/Documentation
Technology
Support
Prescription/Approval
Overall Impression
Login/Registration
Effectiveness
Costs/Reimbursement
Content
2
6
2
3
4
2
5
no data
3
no data
4
1
3
8
3
0
20
40
60
80
100
Percentage
Positiv
Neutral
Negativ

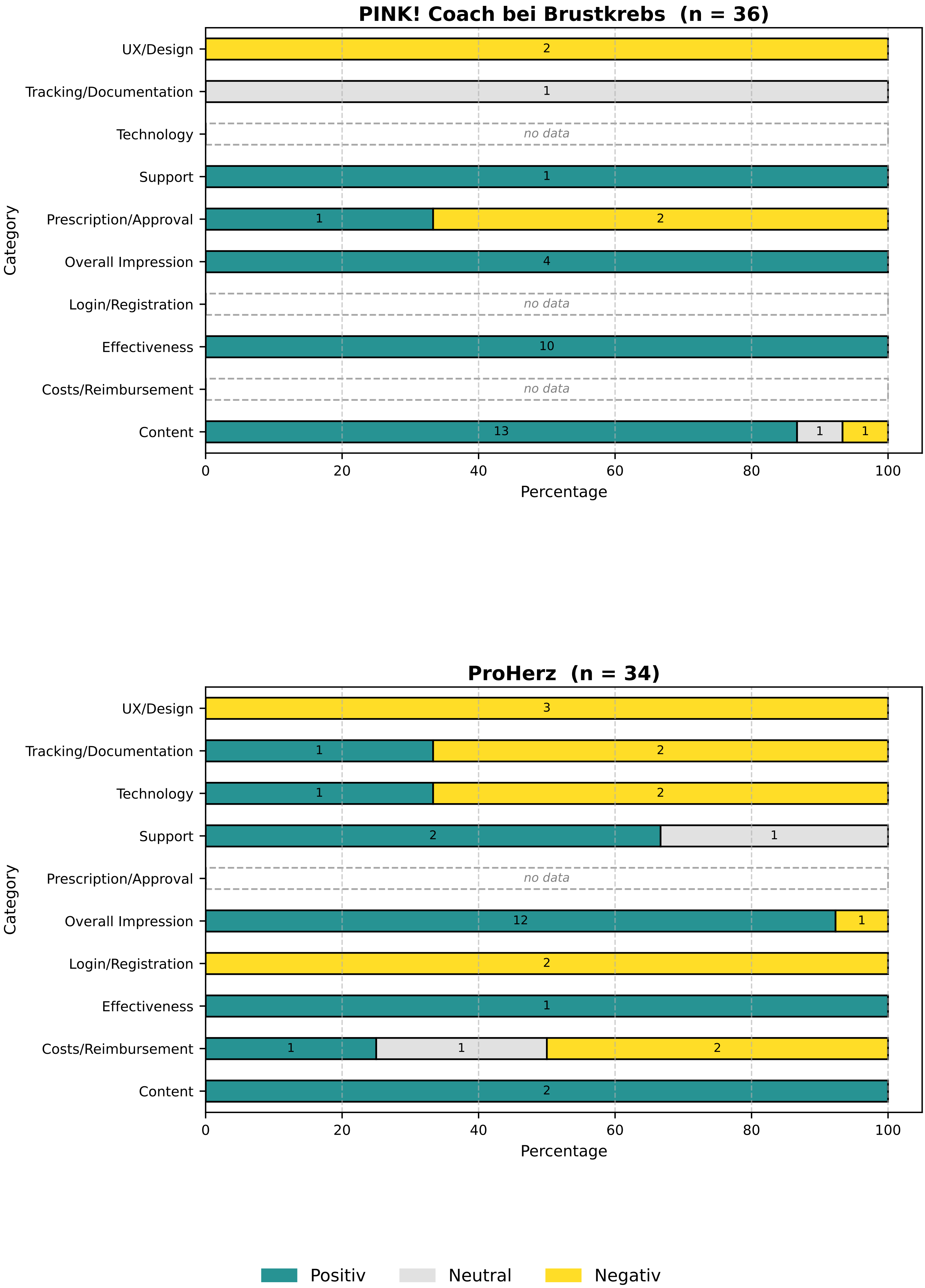

PINK! Coach bei Brustkrebs (n = 36)
UX/Design
Tracking/Documentation
Technology
Support
Prescription/Approval
Overall Impression
Login/Registration
Effectiveness
Costs/Reimbursement
Content
Category
2
1
no data
1
1
2
4
no data
10
no data
13
1
1
0
20
40
60
80
100
Percentage
ProHerz (n = 34)
UX/Design
Tracking/Documentation
Technology
Support
Prescription/Approval
Overall Impression
Login/Registration
Effectiveness
Costs/Reimbursement
Content
Category
3
1
2
1
2
2
1
no data
12
1
2
1
1
1
2
2
0
20
40
60
80
100
Percentage
Positiv
Neutral
Negativ

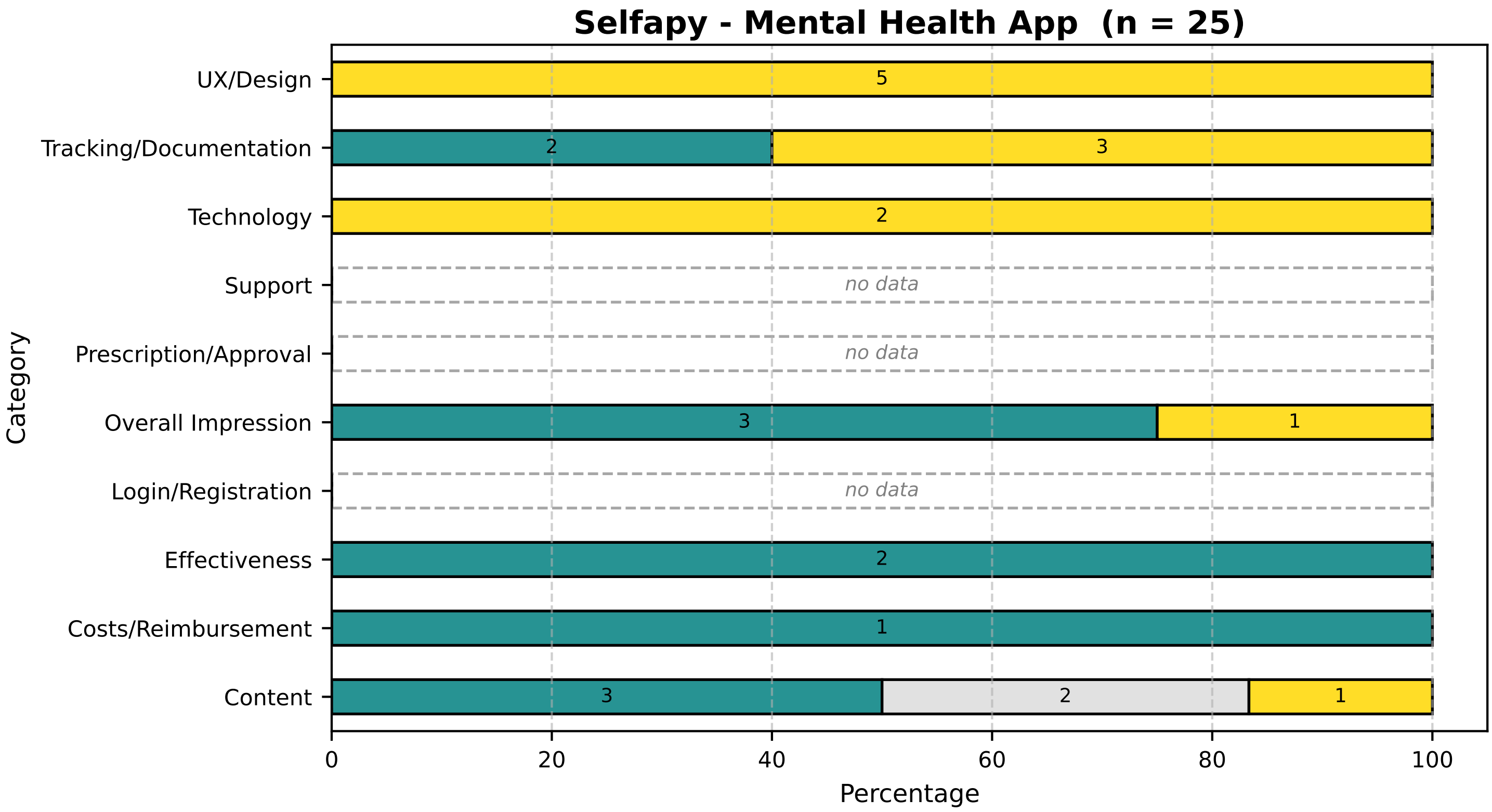
Selfapy - Mental Health App (n = 25)
Category
UX/Design
Tracking/Documentation
Technology
Support
Prescription/Approval
Overall Impression
Login/Registration
Effectiveness
Costs/Reimbursement
Content
5
2
3
2
no data
no data
3
1
no data
2
1
3
2
1
0
20
40
60
80
100
Percentage

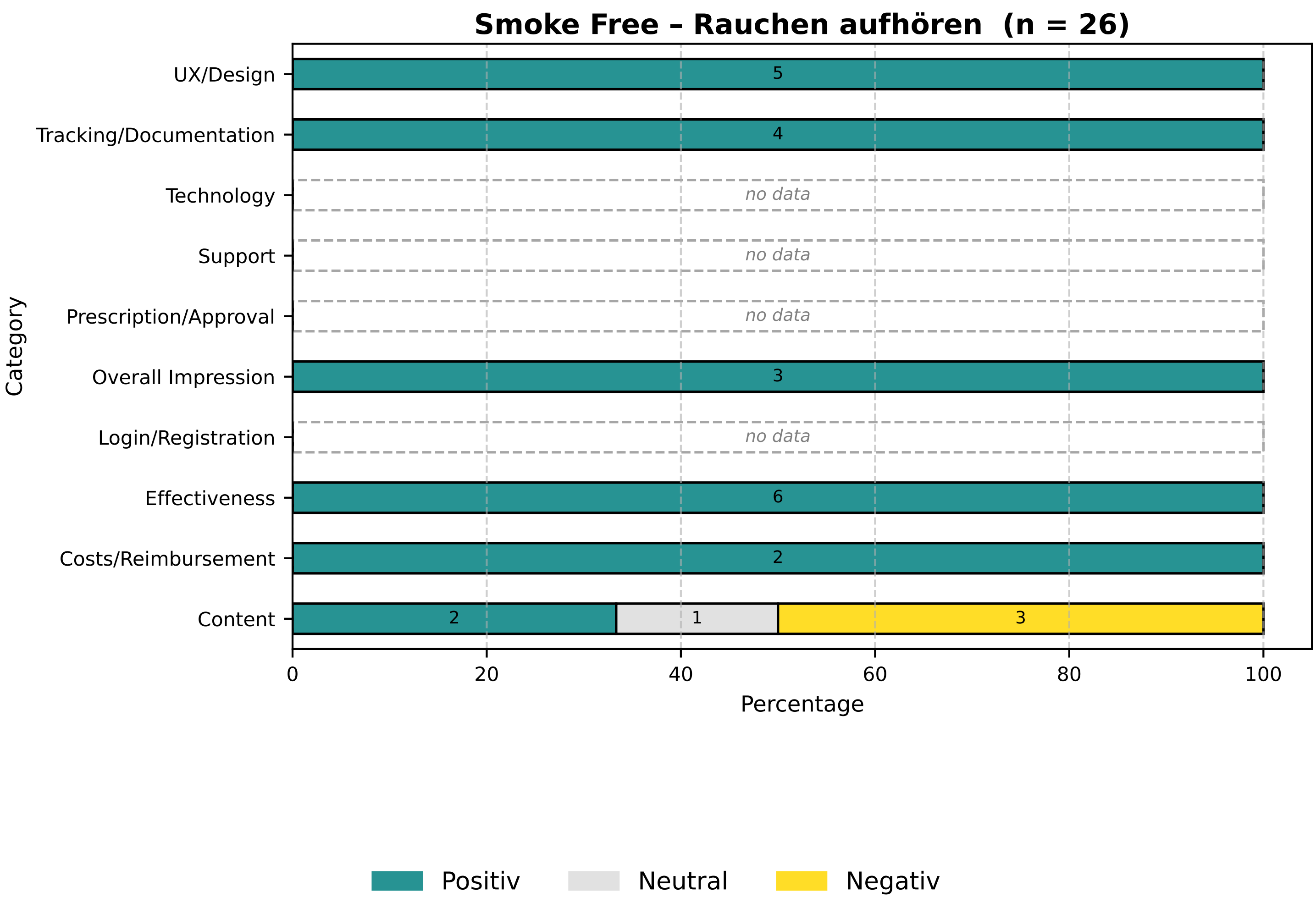
Smoke Free – Rauchen aufhören (n = 26)
Category
UX/Design
Tracking/Documentation
Technology
Support
Prescription/Approval
Overall Impression
Login/Registration
Effectiveness
Costs/Reimbursement
Content
5
4
no data
no data
no data
3
no data
6
2
2
1
3
0
20
40
60
80
100
Percentage
Positiv
Neutral
Negativ

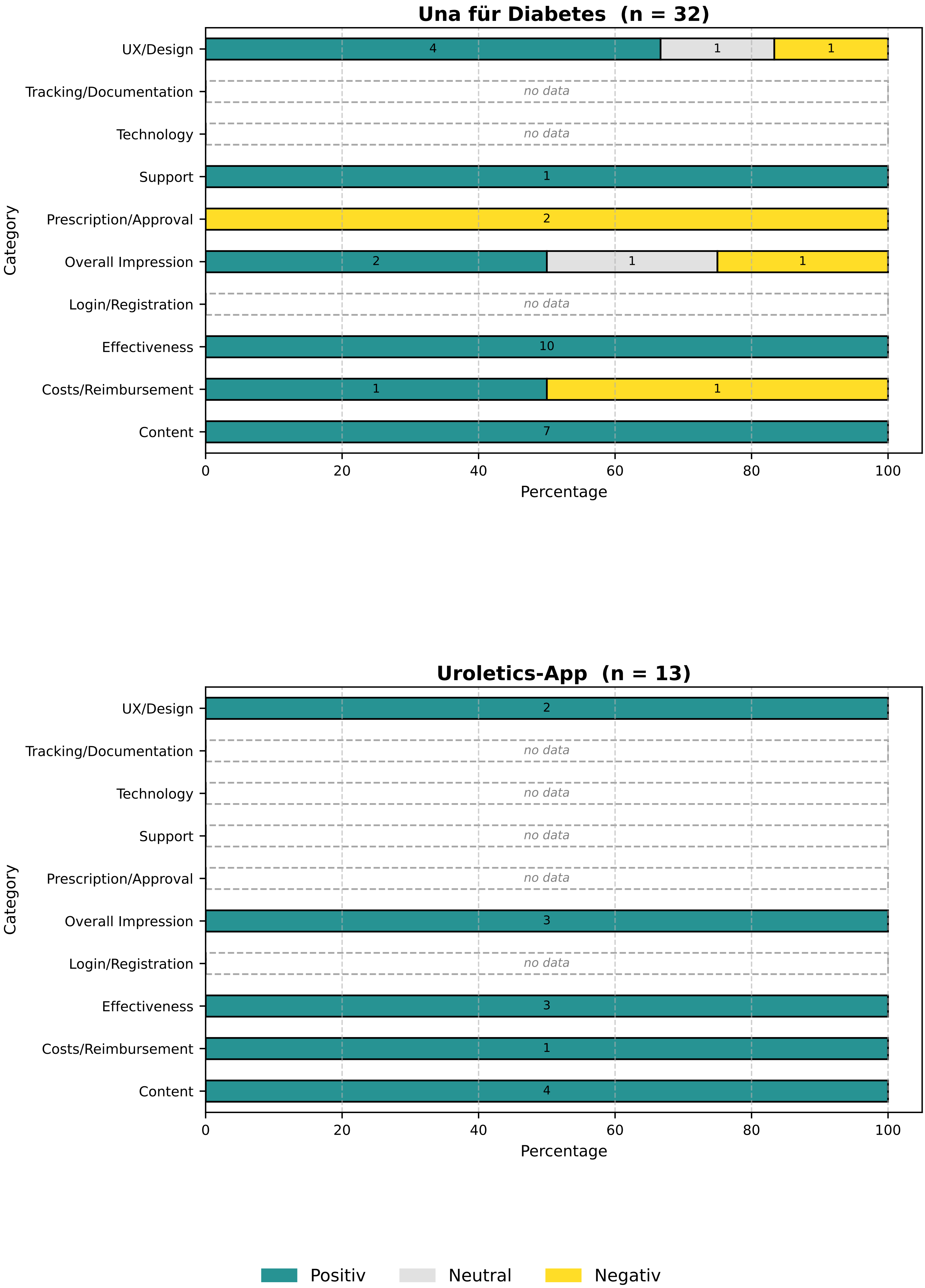

Una für Diabetes (n = 32)
UX/Design
Tracking/Documentation
Technology
Support
Prescription/Approval
Overall Impression
Login/Registration
Effectiveness
Costs/Reimbursement
Content
4
1
1
no data
no data
1
2
2
1
1
no data
10
1
1
7
Category
0
20
40
60
80
100
Percentage
Uroletics-App (n = 13)
UX/Design
Tracking/Documentation
Technology
Support
Prescription/Approval
Overall Impression
Login/Registration
Effectiveness
Costs/Reimbursement
Content
2
no data
no data
no data
no data
3
no data
3
1
4
Category
0
20
40
60
80
100
Percentage
Positiv
Neutral
Negativ

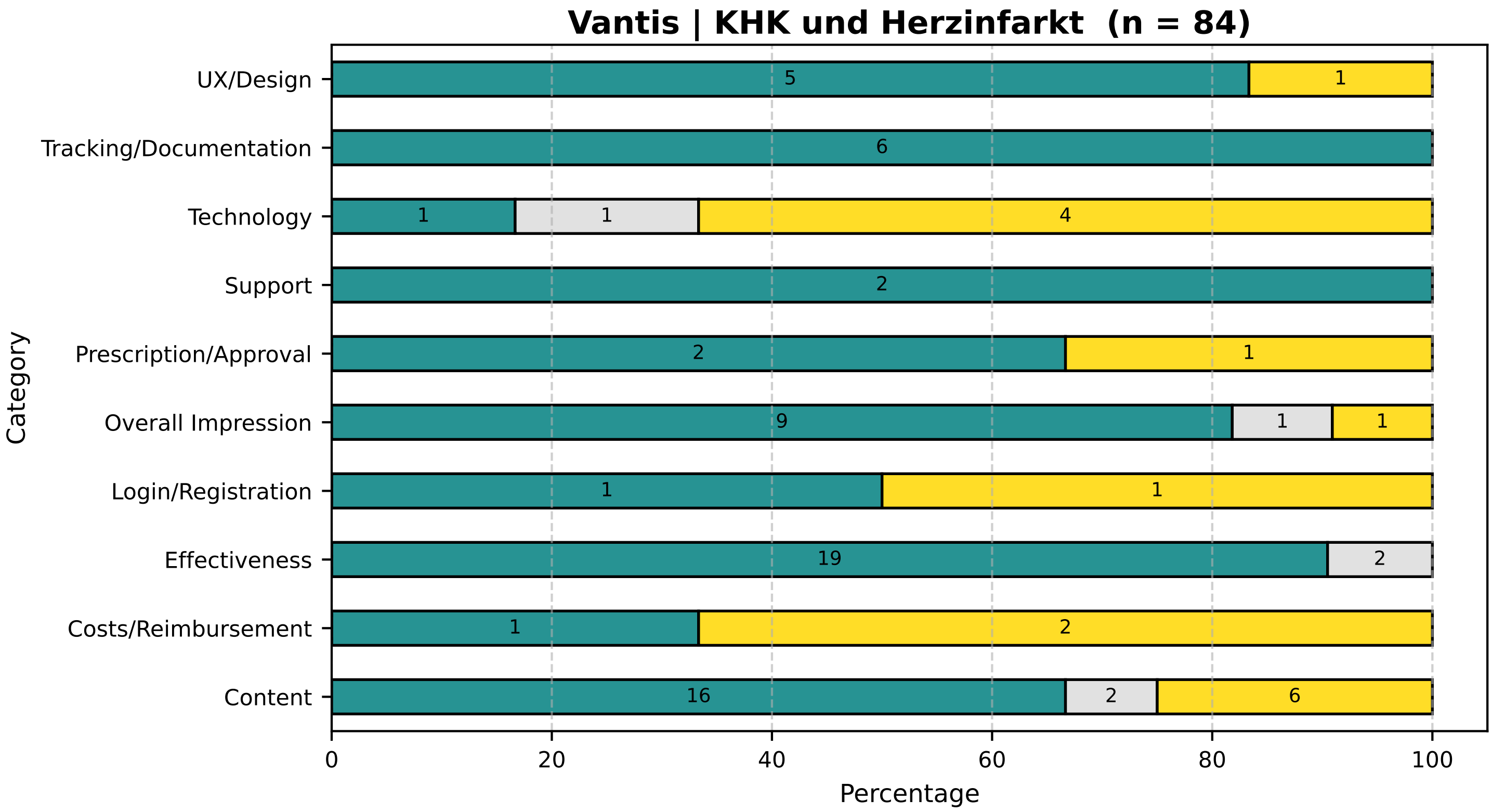
Vantis | KHK und Herzinfarkt (n = 84)
UX/Design
Tracking/Documentation
Technology
Support
Prescription/Approval
Overall Impression
Login/Registration
Effectiveness
Costs/Reimbursement
Content
Category
Percentage
0
20
40
60
80
100
5
1
6
1
1
4
2
2
1
9
1
1
1
1
19
2
1
2
16
2
6

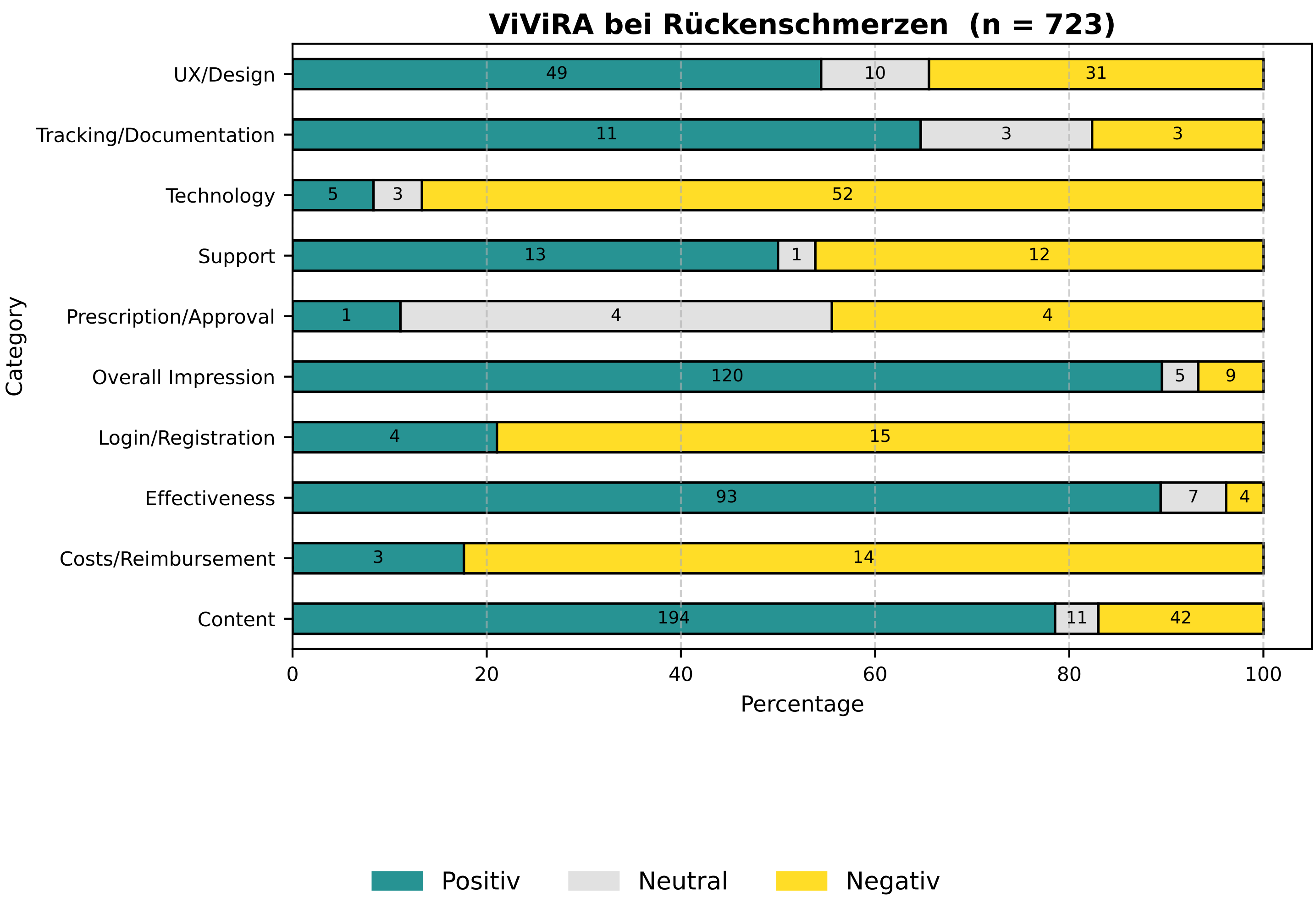
ViViRA bei Rückenschmerzen (n = 723)
UX/Design
Tracking/Documentation
Technology
Support
Prescription/Approval
Overall Impression
Login/Registration
Effectiveness
Costs/Reimbursement
Content
Category
Percentage
0
20
40
60
80
100
49
10
31
11
3
3
5
3
52
13
1
12
1
4
4
120
5
9
4
15
93
7
4
3
14
194
11
42
Positiv
Neutral
Negativ

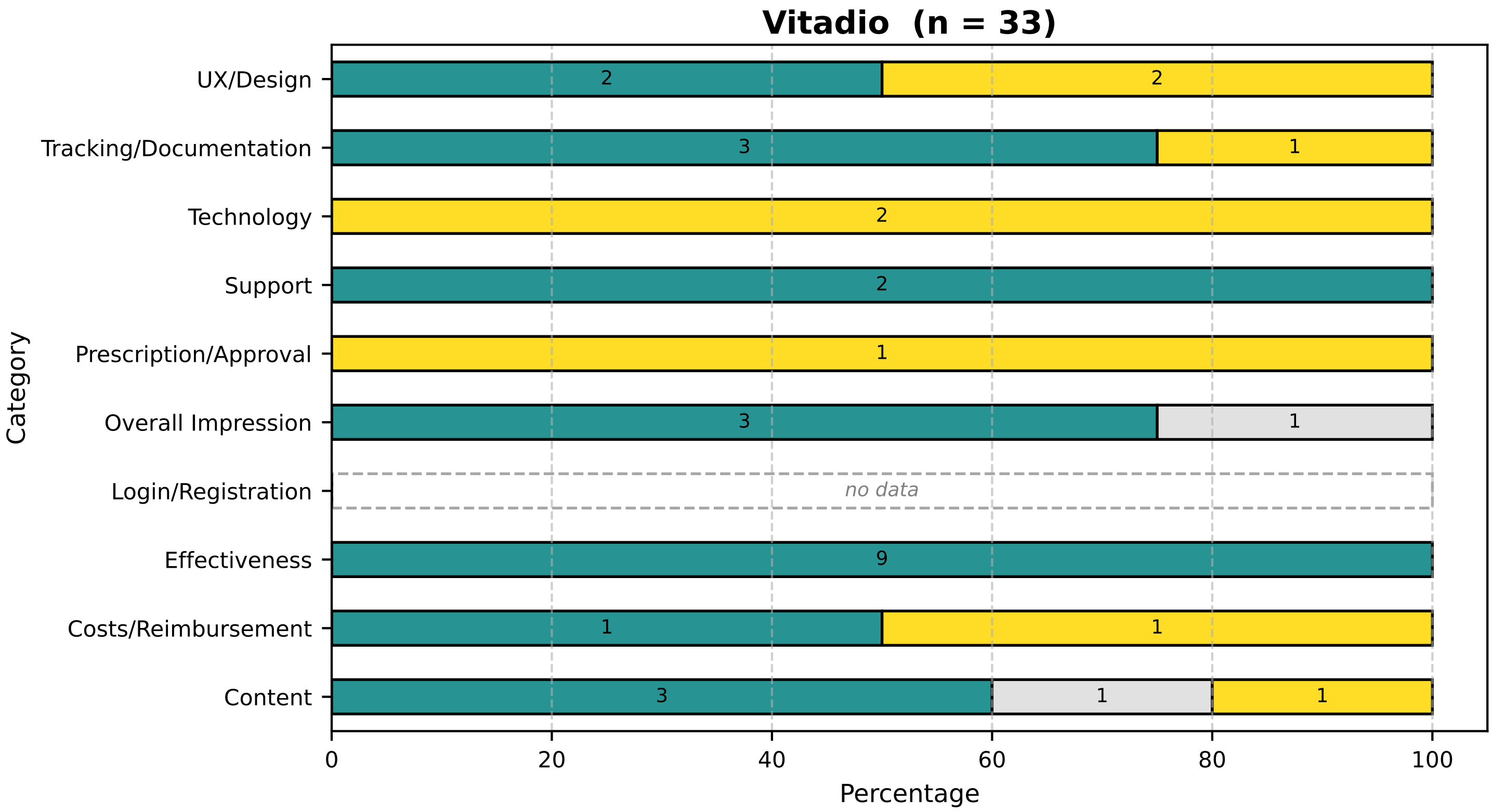
Vitadio (n = 33)
Category
UX/Design
Tracking/Documentation
Technology
Support
Prescription/Approval
Overall Impression
Login/Registration
Effectiveness
Costs/Reimbursement
Content
2
2
3
1
2
2
1
3
1
no data
9
1
1
3
1
1
0
20
40
60
80
100
Percentage

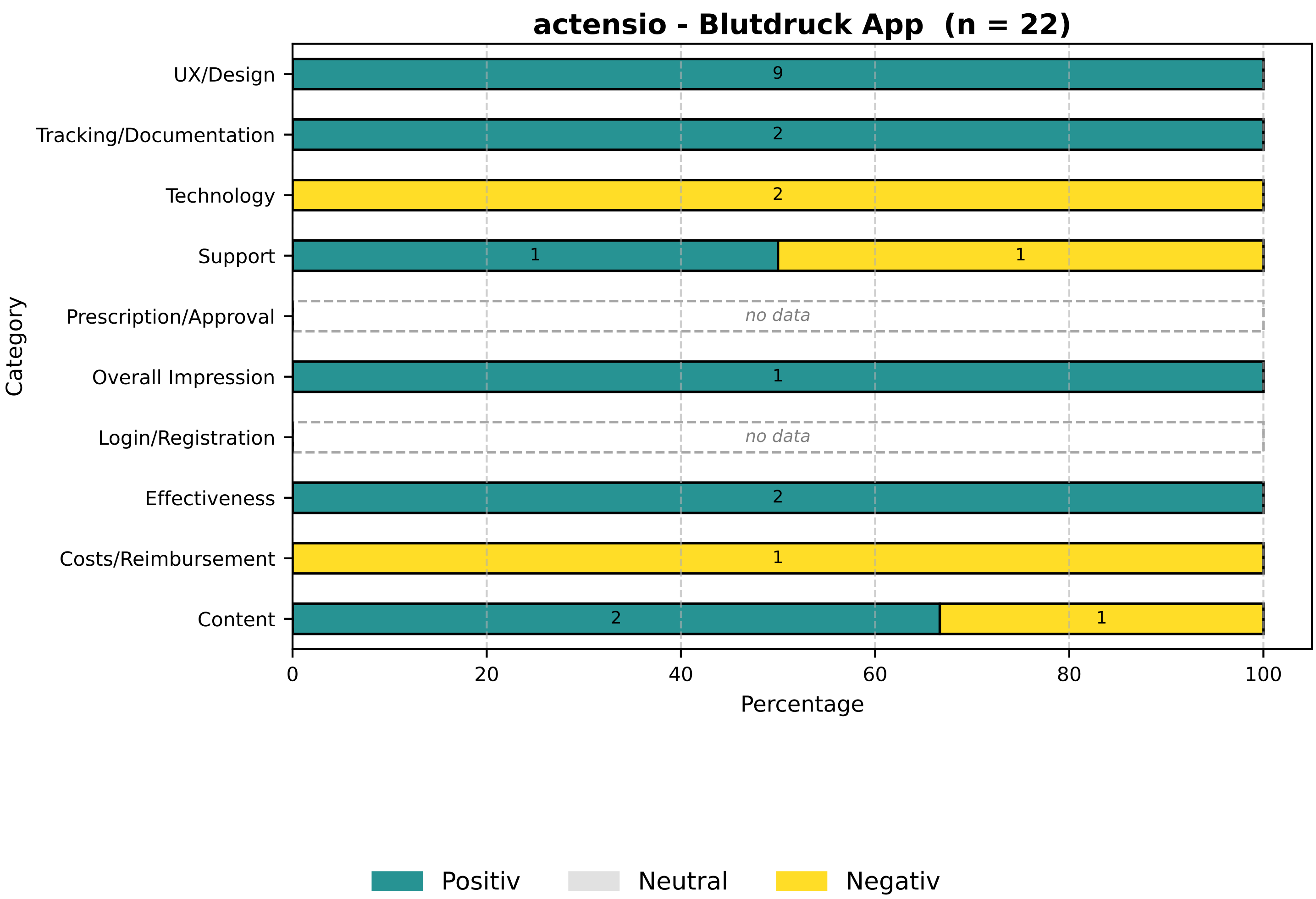
actensio - Blutdruck App (n = 22)
Category
UX/Design
Tracking/Documentation
Technology
Support
Prescription/Approval
Overall Impression
Login/Registration
Effectiveness
Costs/Reimbursement
Content
9
2
2
1
1
no data
1
no data
2
1
2
1
0
20
40
60
80
100
Percentage
Positiv
Neutral
Negativ

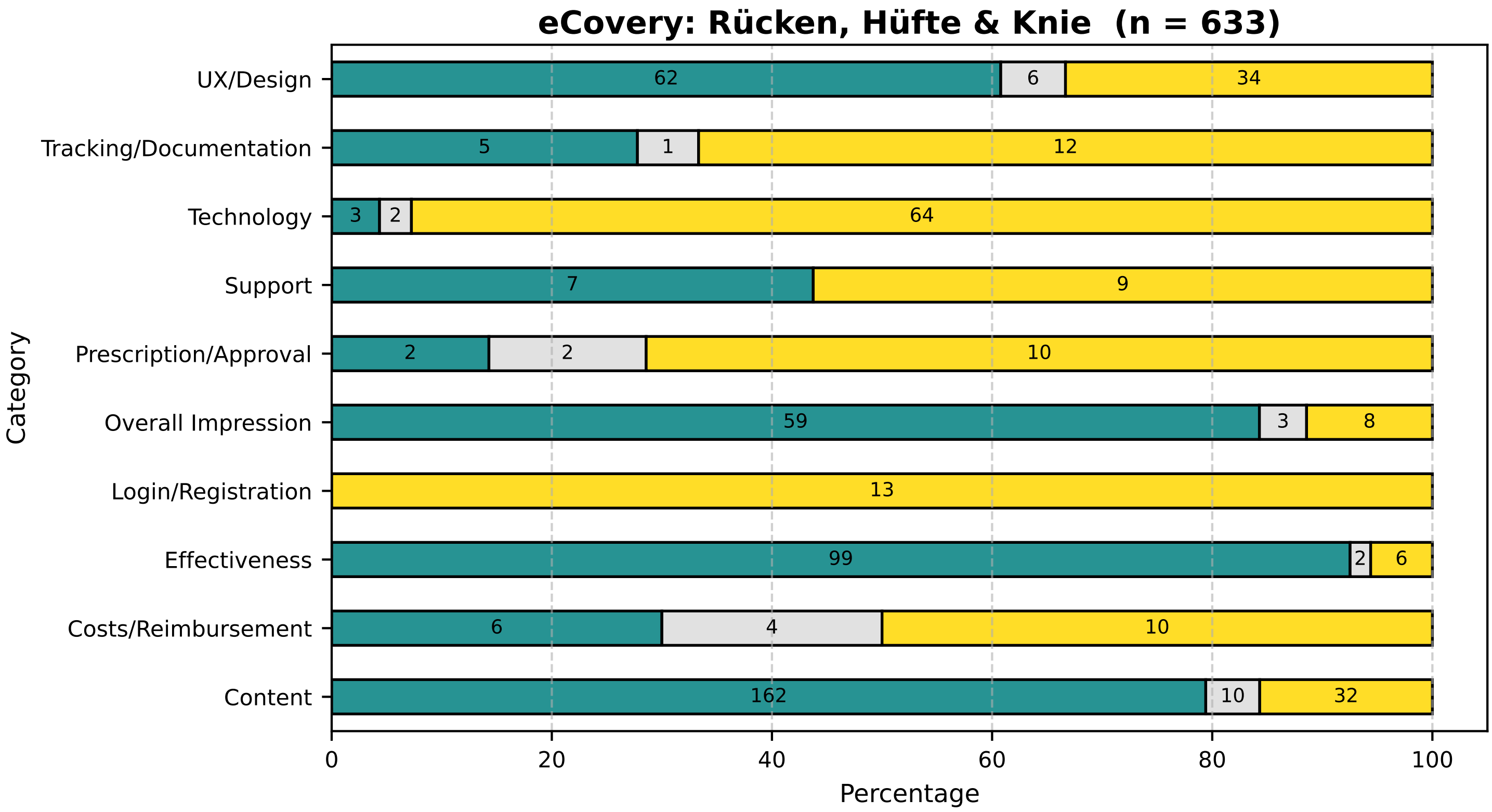

eCovery: Rücken, Hüfte & Knie (n = 633)
UX/Design
Tracking/Documentation
Technology
Support
Prescription/Approval
Overall Impression
Login/Registration
Effectiveness
Costs/Reimbursement
Content
Category
62
6
34
5
1
12
3
2
64
7
9
2
2
10
59
3
8
13
99
2
6
6
4
10
162
10
32
0
20
40
60
80
100
Percentage

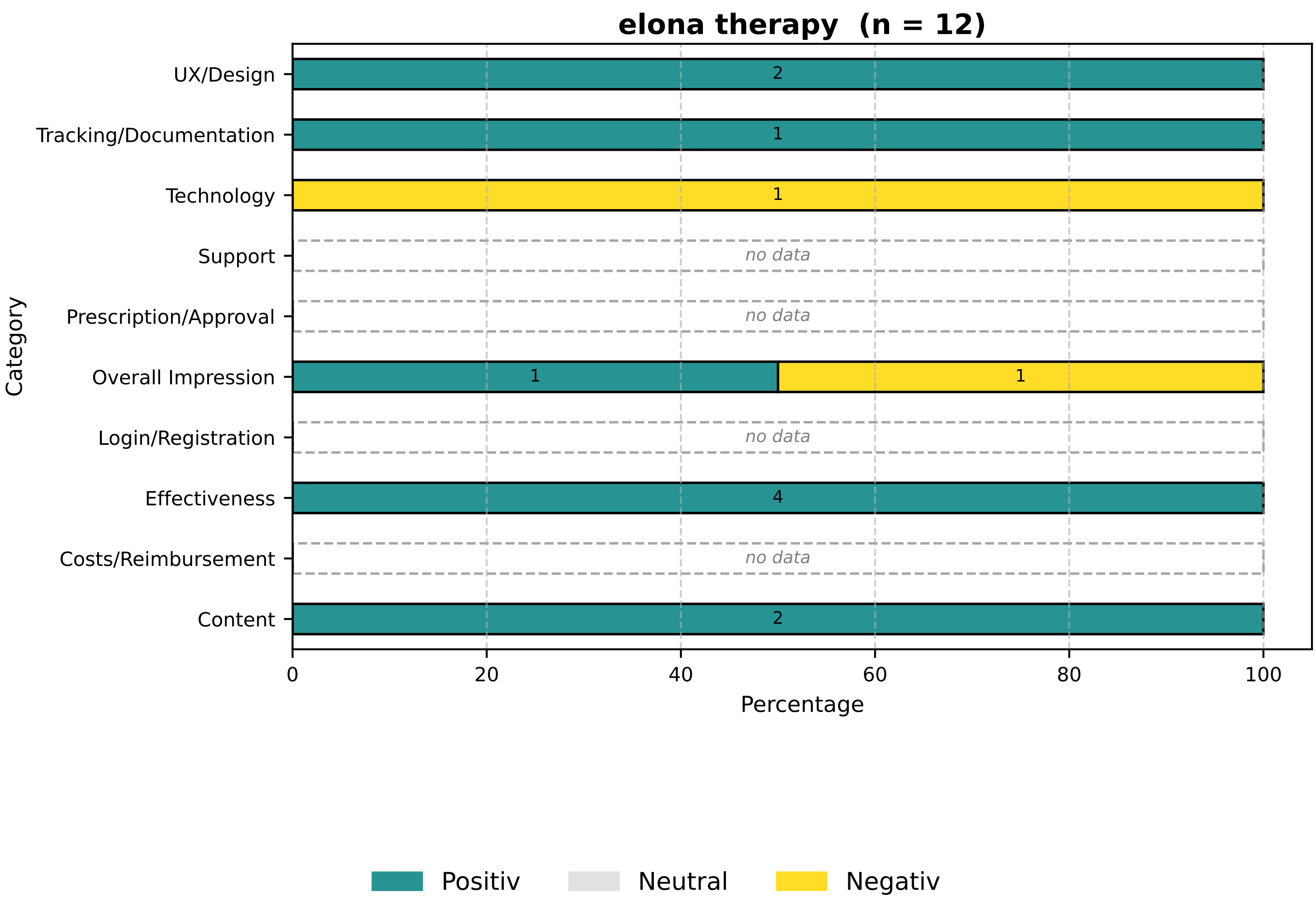

elona therapy (n = 12)
UX/Design
Tracking/Documentation
Technology
Support
Prescription/Approval
Overall Impression
Login/Registration
Effectiveness
Costs/Reimbursement
Content
Category
2
1
1
no data
no data
1
1
no data
4
no data
2
0
20
40
60
80
100
Percentage
Positiv
Neutral
Negativ

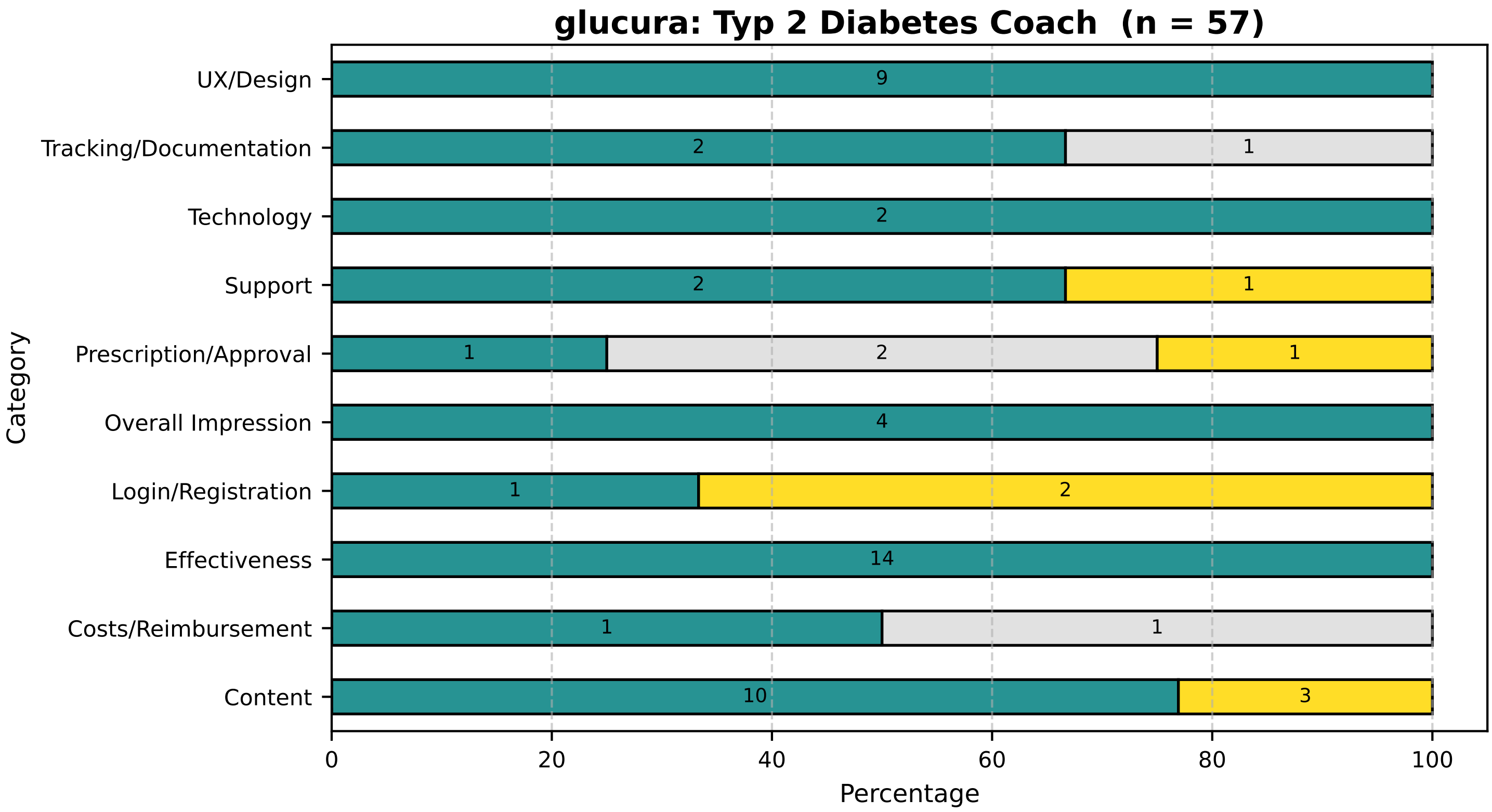
glucura: Typ 2 Diabetes Coach (n = 57)
UX/Design
Tracking/Documentation
Technology
Support
Prescription/Approval
Overall Impression
Login/Registration
Effectiveness
Costs/Reimbursement
Content
Category
Percentage
0
20
40
60
80
100
9
2
1
2
2
1
1
2
1
4
1
2
14
1
1
10
3

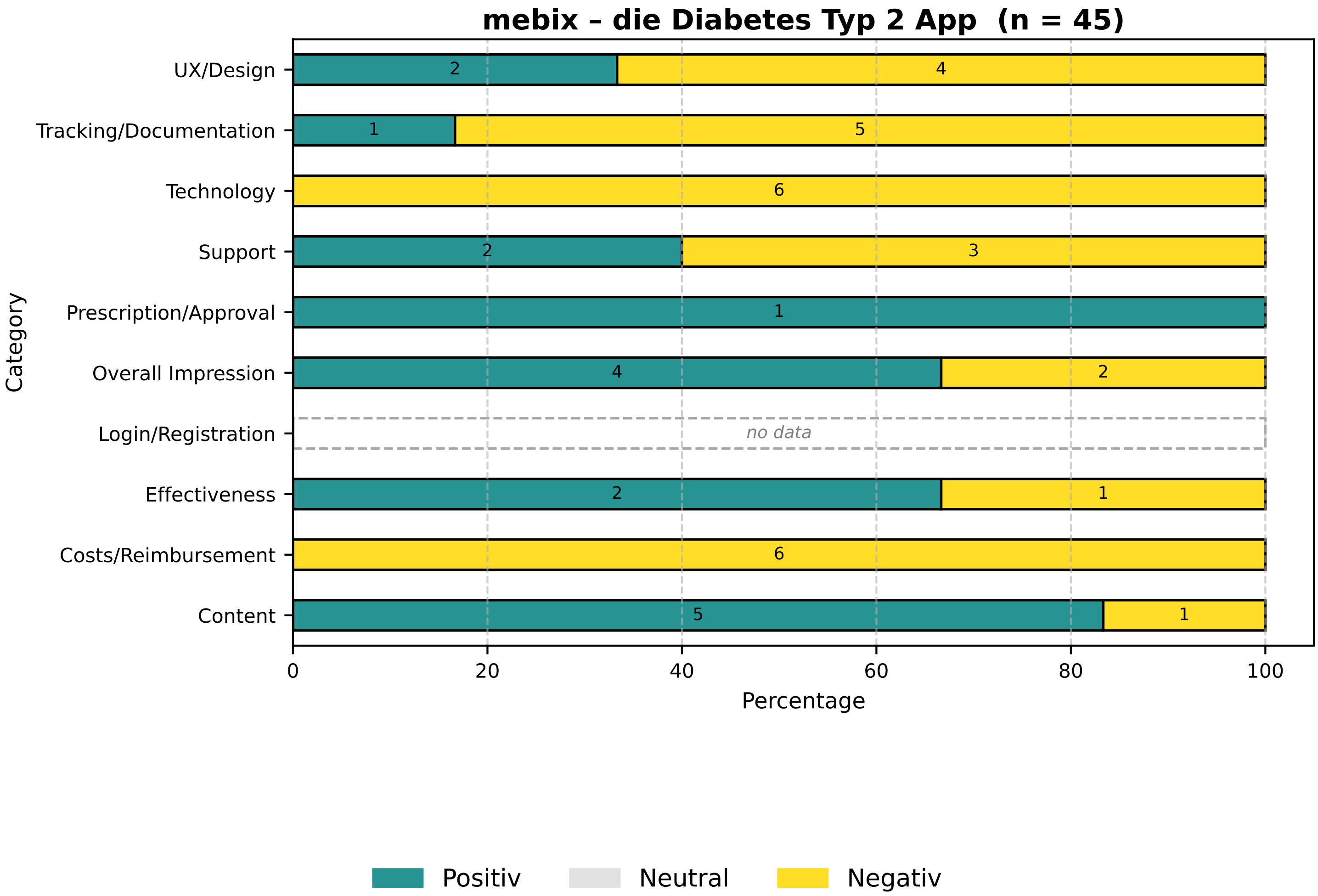
mebix – die Diabetes Typ 2 App (n = 45)
UX/Design
Tracking/Documentation
Technology
Support
Prescription/Approval
Overall Impression
Login/Registration
Effectiveness
Costs/Reimbursement
Content
Category
Percentage
0
20
40
60
80
100
2
4
1
5
6
2
3
1
4
2
no data
2
1
6
5
1
Positiv
Neutral
Negativ

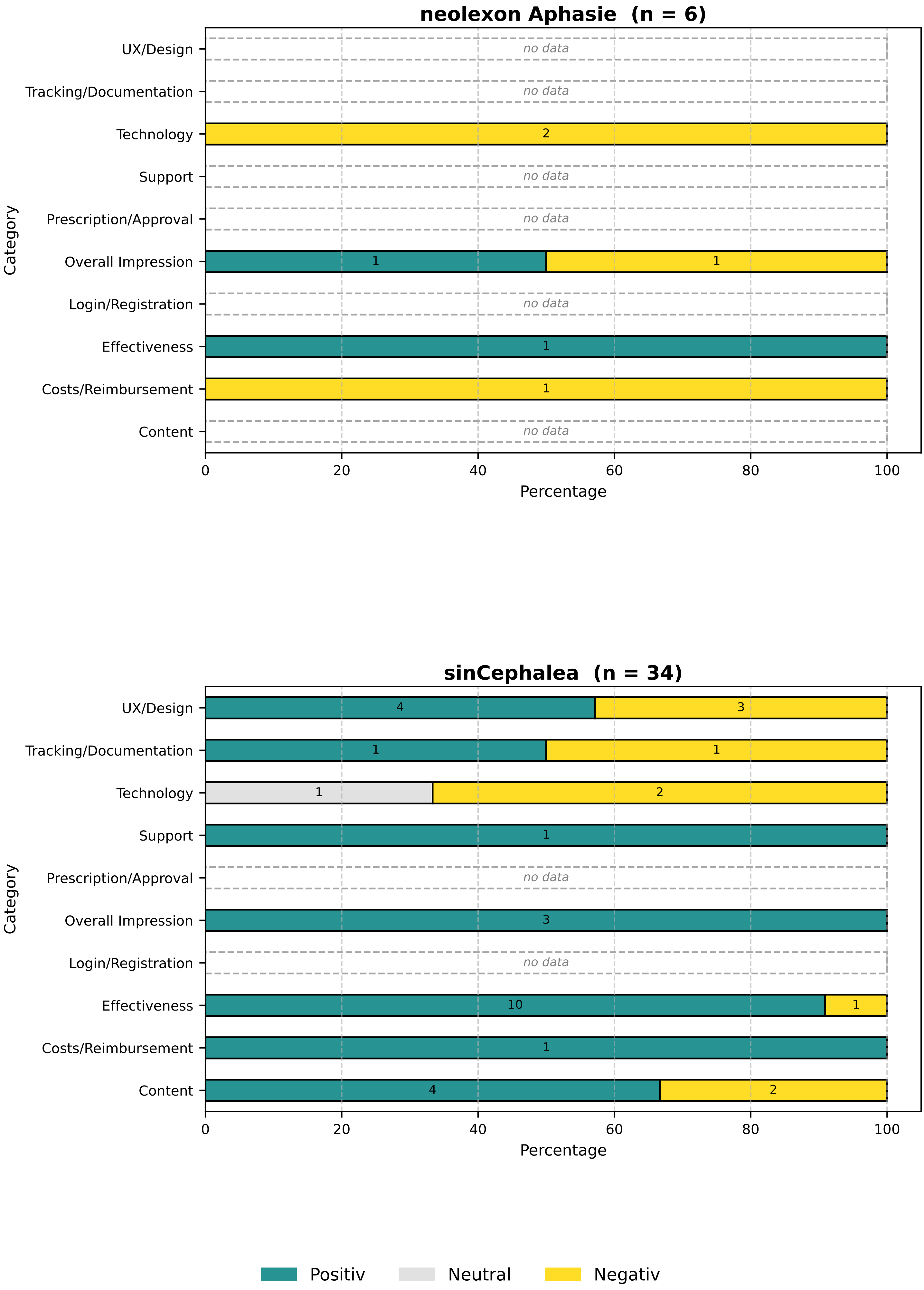

neolexon Aphasie (n = 6)
UX/Design
Tracking/Documentation
Technology
Support
Prescription/Approval
Overall Impression
Login/Registration
Effectiveness
Costs/Reimbursement
Content
no data
2
1
Category
Percentage
0
20
40
60
80
100
sinCephalea (n = 34)
10
3
4
Positiv
Neutral
Negativ

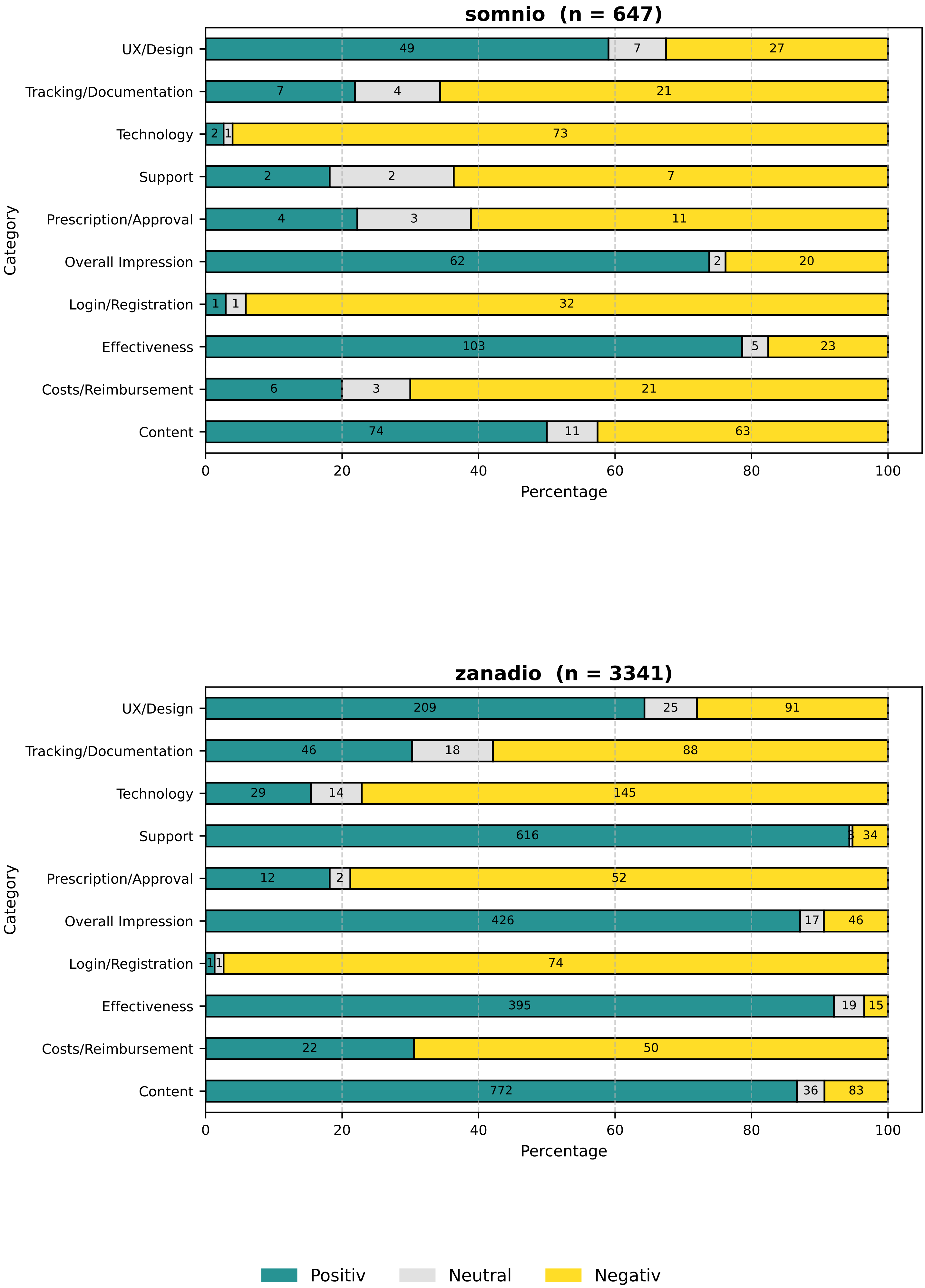

somnio (n = 647)
UX/Design 49 7 27
Tracking/Documentation 7 4 21
Technology 2 1 73
Support 2 2 7
Prescription/Approval 4 3 11
Overall Impression 62 2 20
Login/Registration 1 1 32
Effectiveness 103 5 23
Costs/Reimbursement 6 3 21
Content 74 11 63
Category
0 20 40 60 80 100
Percentage
zanadio (n = 3341)
UX/Design 209 25 91
Tracking/Documentation 46 18 88
Technology 29 14 145
Support 616 34
Prescription/Approval 12 2 52
Overall Impression 426 17 46
Login/Registration 1 1 74
Effectiveness 395 19 15
Costs/Reimbursement 22 50
Content 772 36 83
Category
0 20 40 60 80 100
Percentage
Positiv
Neutral
Negativ
